# Where is my mouth? Rapid experience‐dependent plasticity of perceived mouth position in humans

**DOI:** 10.1111/ejn.14508

**Published:** 2019-07-18

**Authors:** Davide Bono, Patrick Haggard

**Affiliations:** ^1^ Institute of Cognitive Neuroscience University College London London WC1N 3AZ UK

**Keywords:** body ownership, body representation, proprioception, tactile illusion, tactile perception

## Abstract

Several neural and behavioural studies propose that movements of the hand to the mouth are a key motor primitive of the primate sensorimotor system. These studies largely focus on sensorimotor coordination required to reach the mouth with the hand. However, hand‐to‐mouth movement depends on representing the location of the mouth. We report 5 experiments using a novel dental model illusion (DMI) that investigates the neural representation of mouth position. When participants used their right index finger to touch the teeth of an unseen dental model in synchrony with the experimenter's tactile stimulation of the participant's own teeth, participants felt that the position of their own teeth was shifted towards the dental model and stated that their right index finger was touching their actual teeth. This result replicated across four experiments and provides an oral analogue to the rubber hand illusion. Synchrony between the two tactile motions was necessary condition to elicit DMI (Experiment 3). DMI was moderately affected by manipulating the macrogeometric or microgeometric tactile properties of the dental model, suggesting cognitive images of one's own oral morphology play a modest role (Experiments 4 and 5). Neuropsychological theories often stress that hand‐to‐mouth movement emerges early in development or may even be innate. Our research suggests that general, bottom‐up principles of multisensory plasticity suffice to provide spatial representation of the egocentric core, including mouth position.

## Graphical abstract

We report 5 experiments using a novel dental model illusion (DMI) that investigates the neural representation of mouth position. We show that humans can continuously relearn the position of their mouth in space through current sensory tactile inputs received. Our research suggests that general, bottom‐up principles of multisensory plasticity suffice to provide spatial representation of the egocentric core, including mouth position.

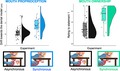

**Abbreviations**
RHI: rubber hand illusion
DMI: dental model illusion

## 1 INTRODUCTION

Moving the hand to the mouth is a basic and primitive motor action, essential for feeding, and thus for survival. This action obviously requires an accurate representation of the position in space of the mouth relative to the hand, implying a form of oral bodily awareness. An important tradition in developmental psychology suggests that the perceived position of the mouth may be specified by a fixed body schema acquired before birth, since accurate hand to mouth coordination emerges early in infancy (by 5 months of age, (Lew & Butterworth, 1997). Indeed, some hand‐to‐mouth behaviours are observed *in utero*, in the 14th–22nd weeks of gestational period (Zoia et al., 2013). Notions of innate representation are bolstered by evidence from clinical neurology of phantom sensation in congenital amelia (Brugger et al., 2000; Melzack, Israel, Lacroix, & Schultz, 1997; Saadah & Melzack, 1994).

Alternative views suggest that correlation between experiences of sensory inputs from the mouth and manual action touches could generate a use‐dependent representation of the position of the mouth in space. Most previous studies of the perceived spatial position of bodily parts have investigated the position of the hand relative to the body (Proske & Gandevia, 2012). Remarkably, few studies have investigated the perceived position of relatively immobile body parts, such as the abdomen, chest and head. Indeed, most discussions of proprioception and body awareness assume that such parts represent a fixed egocentre, used as a reference for defining the positions of more mobile parts, such as hands and feet. As a result, little is known about how perceived spatial position of the mouth is generated, and whether it is fixed or plastic, despite the developmental and evolutionary primacy of the hand‐to‐mouth movement. This paper reports an attempt to fill that knowledge gap.

Tactile illusions have been extensively used in order to study how interactions between multiple sensory inputs produce bodily awareness. In particular, the rubber hand illusion (RHI; Botvinick & Cohen, 1998) investigates how the three‐way correlation between vision, touch and proprioception underlies the feeling that one's body is one's own. In the RHI, visual input showing stroking of the rubber hand captures the perceived location of synchronised stroking on the participant's hand, by a process of visual‐proprioceptive adaptation. This produces a mislocalisation of the participant's hand towards the location of the rubber hand. The shift in the perceived position of one's own hand towards the visual percept due to the visual‐tactile correlation provides a useful quantitative proxy for the illusion that the rubber hand is one's own.

Several studies using the ‘rubber hand illusion’ have suggested that the perceived position of hands (Tsakiris & Haggard, 2005), legs (Crea, D'Alonzo, Vitiello, & Cipriani, 2015), stump (Ehrsson et al., 2008), tongue (Michel, Velasco, Salgado‐Montejo, & Spence, 2014) or even the whole body (van der Hoort, Guterstam, & Ehrsson, 2011) can be adapted by such correlated multisensory experiences. Visual feedback of the body parts is a common feature across all the studies mentioned above, and visual input often dominates bodily awareness (Cardini, Tajadura‐Jiménez, Serino, & Tsakiris, 2013; Fotopoulou et al., 2011; Hagura, Hirose, Matsumura, & Naito, 2012). However, people nevertheless feel a sense of ownership with respect to body parts that are not normally seen, such as the back, the neck (Tipper et al., 1998) and the oral cavity (Fujii et al., 2011). Thus, the sense of one's own body is not defined only by visual experience, although visual inputs often dominate bodily awareness.

Non‐visual paradigms for studying ownership have been proposed, based on a self‐touch version of the rubber hand illusion. In one implementation, the blindfolded participant uses their right hand to stroke a left rubber hand positioned close to their midline. At the same time, the experimenter strokes the participant's left hand, which lies on the table in front of the left shoulder (Ehrsson, Holmes, & Passingham, 2005, figure 1). The synchronised tactile and proprioceptive input adapt the perceived position of the left hand, resulting in the familiar ‘proprioceptive drift’ in which the left hand is perceived as shifted towards where the left rubber hand is seen, rather than where the participant's left hand actually is. In addition, participants report a feeling of ownership with respect to the rubber hand (Ehrsson et al., 2005). This tactile‐proprioceptive method is suitable for probing the sense of ownership over body parts that are not normally seen. However, to our knowledge, it has been used only to study perception of *limb* position, and not of other body parts (Aimola Davies, White, & Davies, 2013).

Here, we applied tactile, non‐visual stimulation to manipulate body ownership with respect to the upper teeth. Our main research question consists of investigating whether synchronous tactile‐proprioceptive experience could induce plasticity in representation of the teeth. Several reasons motivate focussing on the teeth and mouth in studying body ownership: these structures are not experienced visually, yet they are often considered part of a human egocentre, or stable reference point within the body representation (Alsmith & Longo, 2014). Finally, the mouth has often been considered as a fixed, even innate element of body representation (Slater, 1999). The idea of innate representation underlying bodily experience receives support from reports of phantom pain in persons with congenital absence of a limb (Gallagher, Butterworth, Lew, & Cole, 1998; Makin & Bensmaia, 2017; Melzack, 1990). However, other studies have highlighted the role of sensory experience in body representation by showing that blind individuals have a less dynamic multisensory representation of their own limbs (Nava, Steiger, & Röder, 2014; Petkova, Zetterberg, & Ehrsson, 2012).

The hand to mouth movement is often described as an innate sensorimotor primitive, because it appears immediately after, or even before birth. Moreover, the hand to mouth action appears both well‐formed and goal‐directed, in contrast to babbling movements of neonates (Butterworth & Hopkins, 1988; Gallagher, 2006). This movement would then be the archetype or starting point for all other skilled actions, and indeed for wider cognition (Piaget, 1952). Clearly, hand‐to‐mouth movements require an accurate spatial representation of the position of the mouth. This view would imply that experience‐dependent plasticity of spatial representation, which is so characteristic of the brain's hand representation, should be entirely absent for the mouth.

To investigate this hypothesis, we designed several independent experiments to investigate multiple research questions. We first investigated whether correlated tactile and proprioceptive experience are sufficient to elicit changes in the perceived location and sense of ownership with respect to the teeth and the mouth. Accordingly, we manipulated the spatial and temporal aspects of tactile stimulation in Experiments 1 and 2, and spatial aspects only in Experiment 3. Second, we investigated whether mouth stimulation causes experience‐dependent modulation of the mouth only or also of other body parts. Consequently, in Experiments 1 and 3 we asked to judge the perceived position of both stimulated and adjacent non‐stimulated body parts. Last, we wanted to understand to what extent top‐down knowledge (Tsakiris & Haggard, 2005) about the morphology of one's own teeth influence any experience‐dependent plasticity effects. To address this research question, we modified the microgeometric (Experiment 4) and macrogeometric aspects (Experiment 5) of the dental model used during tactile stimulation.

## 2 MATERIALS AND METHODS

### 2.1 Participants

In total, 48 volunteers (33 female, aged 18–39 years of age) were recruited from the Institute of Cognitive Neuroscience subject data pool for six separate experiments. All the participants gave their informed written consent prior to participation, and they were naïve as regarding to the actual purpose of the experiment. All the volunteers recruited were healthy adults without neurological or psychiatric history, non‐smokers, without dental hypersensitivity, history of previous traumatic teeth injuries or any dental treatments during the preceding year. Experimental design and procedure were conducted in accordance with principles proposed in the Declaration of Helsinki and were approved by the UCL research ethics committee.

### 2.2 The dental model illusion

In Experiments 1–5, the dental model illusion paradigm was used as an explicit measure to study proprioception and the sense of teeth ownership. In each experimental session, participants rested their arms on the table and their head on a chinrest. Participants were then blindfolded to eliminate any visual input and remained blindfold throughout the experiment.

**Figure 1:**
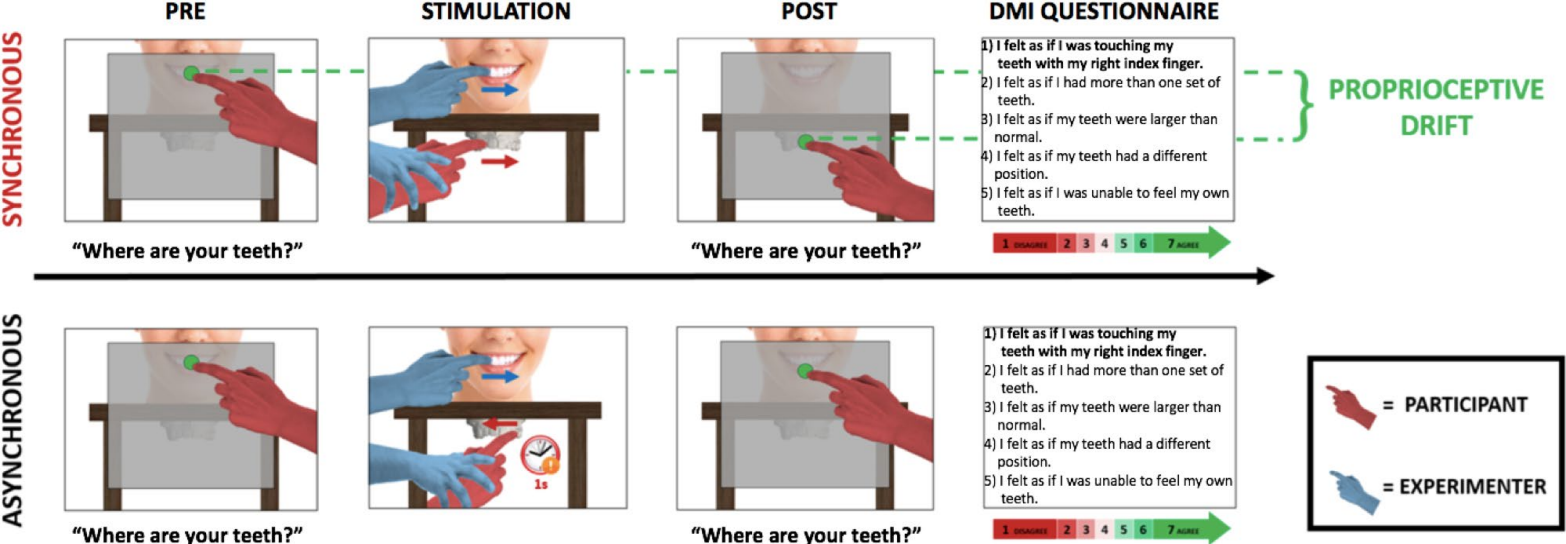
Structure of a single trial of the dental model illusion (DMI) paradigm. Each trial comprises four phases: pre‐test perceived position estimation of teeth, tactile stimulation, post‐test perceived position estimation of teeth and questionnaire. In the tactile stimulation, there are two stroking conditions: synchronous and asynchronous. During the synchronous condition, the experimenter guides the participant's right index finger to slide over the facial surface of the maxillary central and lateral incisors of a dental model placed 8 cm beneath the chinrest. Simultaneously, the experimenter touches with their index finger the facial surface of the participant's central and lateral maxillary incisors. The direction of the two touches is identical. During the asynchronous condition, there is a 1‐s delay in between the two touches, and the direction of the two touches is therefore different. [Colour figure can be viewed at http://www.wileyonlinelibrary.com]

Participants were told that during each trial, the experimenter would passively guide the participant's right index finger to touch and move across their own central and lateral maxillary incisors. Each trial was composed by four phases: pre‐test position estimation, tactile stimulation, post‐test position estimation and a questionnaire (see figure 1). A pre‐test estimate of the position of the maxillary central incisors was obtained, as follows. Participants were instructed to judge the position of their maxillary central incisors (Experiments 1, 2, 3, 4 and 5) and, as a second control judgement, in Experiments 1 and 3 only, of the tip of their nose, by projecting a parasagittal line from the tooth/nose to a wooden board placed 5 cm in front of their face in the frontoparallel plane. They were asked to point with their left index finger to the position on the board immediately in front of the location of the designated body part. This task is directly based on pointing tasks measuring limb position sense (Jola, Davis, & Haggard, 2011; Longo & Haggard, 2010). The endpoint of each pointing movement on the board was measured with a ruler by the experimenter. In Experiments 1 and 3, the order of nose/teeth pointing trials was randomised across trials.

**Figure 2:**
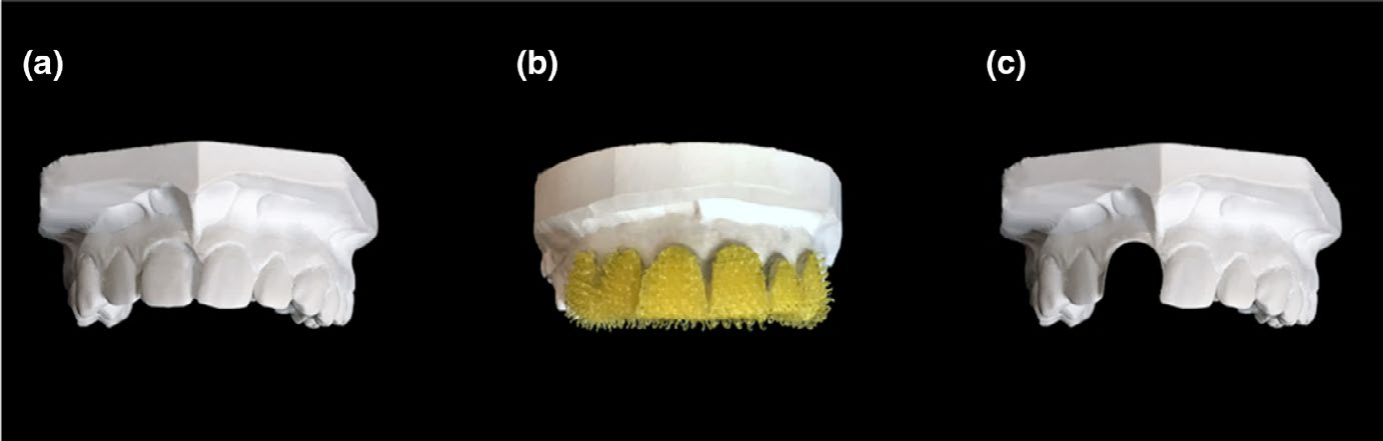
Dental models of the maxillary dental arch used in the DMI paradigm. Plaster dental model with a complete set of teeth and texture comparable with the human dentition (a), plaster dental model with central and lateral incisors covered with ‘spiky’ Velcro (Sellotape^®^) (b), plaster dental model with the right central incisor removed (c). [Colour figure can be viewed at http://www.wileyonlinelibrary.com]

At the end of the pre‐test pointing phase, the board to which participants pointed was removed and the tactile stimulation of participant's teeth started. Both the participant and the experimenter wore identical sterile latex‐free gloves in their right hands to make the tactile feel of the two hands as similar as possible (Ehrsson et al., 2005). Before each tactile stimulation, the experimenter applied a repetitive supination/pronation movement to the participant's right forearm, to reset proprioceptive drift and minimise carry over between trials (Kapandji, 2001; Kisner & Colby, 2002; Kwon et al., 2013; Sanes & Evarts, 1984). The tactile stimulation phase of each trial lasted 60s and consisted of two gentle stroking movements, as follows. One movement involved *participant‐to‐model touch*. The experimenter guided the participant's right index finger to slide over the facial surface of the central and lateral maxillary incisors of a dental model made of plaster placed beneath the chinrest. The distance between the participant's maxillary incisors and the incisors of the dental model was 8 cm. The other movement involved *experimenter‐to‐participant touch*. The experimenter slid his own index finger over participant's central and later maxillary incisors. At the end of each stroke, the experimenter's finger was removed from participant's teeth to return to the starting point through air. The same applied for the participant's index finger. The stroking direction, from left to right or right to left, was randomised. The pressure and velocity exerted for the two touches were kept as constant as possible by the experimenter. The dental model used for the stimulation was the same for each participant (see Figure 2). After stimulation, participants made a post‐test estimation of the position of both their central maxillary incisors (Experiments 1, 2, 3, 4 and 5) and nose (Experiments 1 and 3). The procedure applied was identical to the one used for the pre‐test estimate.

At the end of each trial, participants completed a ‘Dental Model Illusion Questionnaire’. This questionnaire, based on the one used by Botvinick and Cohen (1998), was composed of five statements referring to their perceptual experiences during stimulation, each rated on a 7‐point scale. Only the first statement, ‘*I felt as if I was touching my teeth with my right index finger*’, was designed to correspond to the illusion. The other four statements (see Figure 1) were unrelated to the illusion. They were used as control statements for suggestibility. A list of all the statements of the dental model illusion (DMI) questionnaire can be found in the supporting information section.

### 2.3 Experiments 1, 2 and 3: spatio‐temporal pattern of tactile stimulation across body parts

We first investigated whether the effect of the DMI is local and specific to the stimulated teeth or if it extends also to other body parts. For this reason, in both experiments 1 and 3, baseline pre‐test judgement prior to stimulation and post‐test judgement after stimulation were obtained not only for the central maxillary incisors but also for the tip of the nose. These experiments also aimed to understand the forms of stroking that elicit the dental model illusion. Experiment 2 was a manipulation check to investigate whether the DMI is sensitive to the specific parameters of stroking in the asynchronous and synchronous conditions.

**Figure 3:**
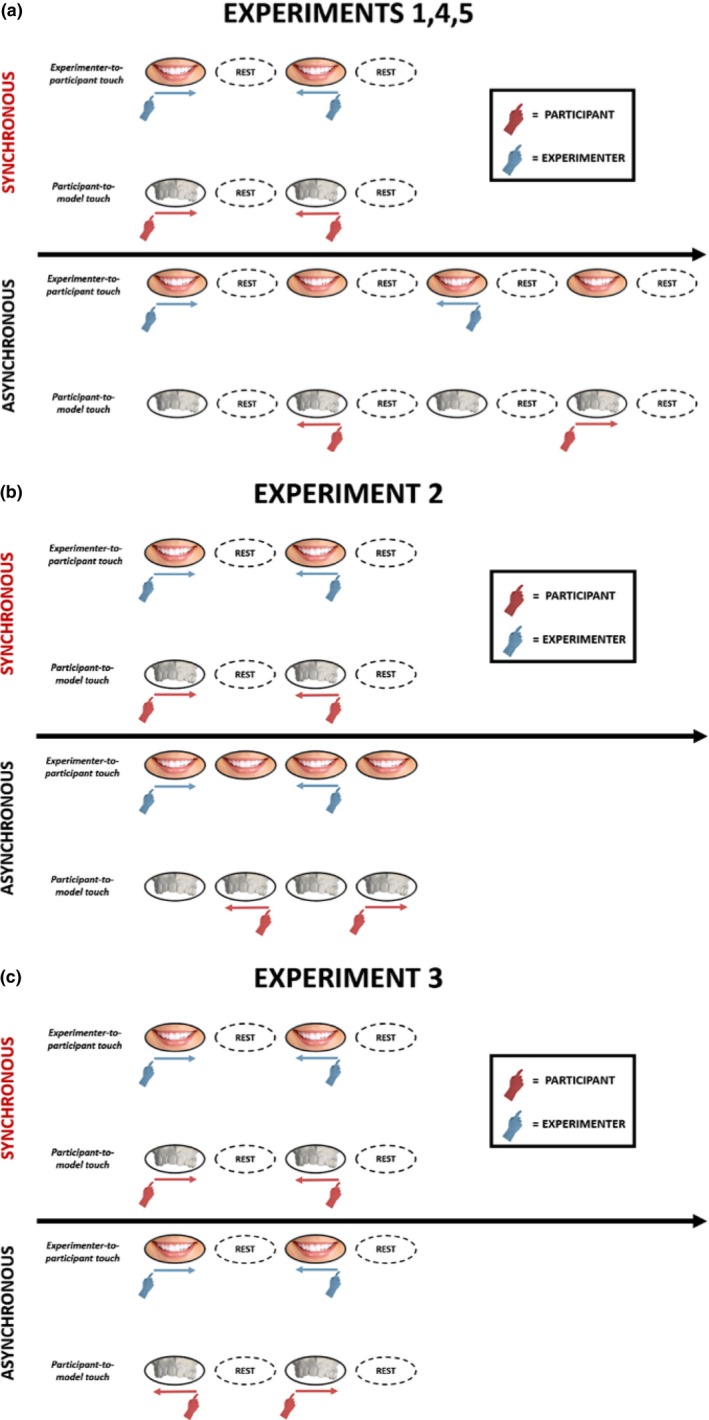
Structure of the tactile stimulation phase of the DMI paradigm across experiments. In Experiments 1, 4 and 5, the overall amount of tactile inputs across different stroking conditions was different. In the synchronous condition, each cycle lasted 4 s (frequency 0.25 Hz). In the asynchronous condition, each cycle lasted 8 s (frequency 0.125 Hz). This arrangement was preferred because it made the asynchrony highly perceptually salient for the participant and helped the experimenter to deliver the most accurate stroke timing possible (a). In Experiment 2, the length of a stimulation cycle was equal to 4 s in both the stroking conditions (frequency 0.25 Hz) (b). In Experiment 3, the length of a stimulation cycle was equal to 4 s in both the stroking conditions (frequency 0.25 Hz). However, in this experiment only the spatial aspects of the stroking factor were manipulated. [Colour figure can be viewed at http://www.wileyonlinelibrary.com]

In Experiment 1, participants (*N* = 8, 6 females, mean age ± SD: 22.9 ± 1.6 years) experienced 9 trials of synchronous and 9 trials of asynchronous stroking. In the absence of any previous experiments on the perceived position of the mouth, we had no established estimate of effect size. Therefore, the effect size was estimated from a pilot experiment (*N* = 66) conducted during a public science demonstration held at Tate Modern, London, in April 2017. In the pilot, we observed a drift towards the model in the perceived position of the maxillary central incisors due to synchronous stroking of teeth, with a Cohen's dz of 1.27. For sample size calculation, we assumed a two‐tailed 5% type I error rate and 80% power. The pilot did not involve any asynchronous stroking condition, whereas the key effect in the experimental studies was the difference in drift between synchronous and asynchronous conditions. Pilot testing consisted of three phases: pre‐test position estimation, 60s of synchronous tactile stimulation and post‐test position estimation. The procedure used during these three phases was identical to Experiments 1–5. The power calculation, conducted with G*Power (Faul, Erdfelder, Lang, & Buchner, 2007), suggested that seven participants were required to demonstrate the effect. However, we chose a minimum sample size of 8. As in previous RHI studies, our experimental design compared synchronous and asynchronous stroking conditions. In the synchronous stroking condition, each stimulation cycle lasted 4 s (Frequency = 0.25 Hz). First, the participant's teeth and the dental model were stroked simultaneously, and with the same direction of motion (see Figure 3a). The stroke lasted for 1 s. This was followed by 1‐s rest without stimulation, during which the experimenter's finger was removed from the participant's teeth, and the participant's finger was removed from the dental model. Next, the stroking was repeated in the reverse direction, again lasting 1 s. This was followed by a further 1 s with no stimulation, after which the cycle was repeated.

In the asynchronous condition, each stimulation cycle lasted 8 s (frequency = 0.125 Hz) and consisted of eight phases, as follows. The experimenter stroked the participant's teeth (1 s), rest with no stimulation (1 s), the participant stroked the model (1 s), rest with no stimulation (1 s), the experimenter stroked the participant's teeth in the reverse direction (1 s), rest with no stimulation (1 s), and the participant stroked the model in the reverse direction (1 s), rest with no stimulation. This arrangement was preferred because it made the asynchrony highly perceptually salient. The order of synchronous/asynchronous trials was randomised across the experiment for each participant. However, given that stroking always lasted for a fixed period of 60 s, it resulted in more overall stimulation during the synchronous than the asynchronous condition.

Experiment 2 (*N* = 8, six females, mean age ± SD: 23 ± 2.9 years) used a different form of asynchronous stimulation, which exactly balanced the overall amount of tactile input across the different stroking conditions. To do this, the frequency of stimulation used in the asynchronous condition was equal to the frequency used in the synchronous condition. Both the synchronous and the asynchronous stimulation cycles lasted 4 s. This goal was achieved by removing the 1‐s rest between successive touches in the asynchronous conditions (see Figure 3b). All the other aspects of the method were equal to Experiment 1.

Participants in Experiments 1 and 2 gave their consent to be filmed during testing. Frame‐by‐frame analysis of the video record allowed us to verify the timings of manual stimulation. For both the experiments, 10 trials of asynchronous stroking condition were randomly selected. Within each randomly selected trial, a random cycle of stimulation was selected, and two random consecutive touches were then randomly identified. We therefore calculated the inter‐movement delay as the delay in time between the offset of the first randomly selected touch and the onset of the next. In Experiment 1, the average of the 10 inter‐movement delays was equal to 0.978 ± 0.060 s, whereas in Experiment 2, it was equal to 0.173 ± 0.185 s. This analysis confirms that manual stimulation in Experiments 1 and 2 broadly followed the design plan.

In Experiment 3 (*N* = 8, six females, mean age ± SD: 21.7 ± 2 years), only the spatial congruency/incongruency of stroking was manipulated. The frequency of stimulation was equal across stroking conditions, and stroking was always temporally synchronous. The direction of motion on participant's teeth and the dental model was modulated so that the two simultaneous stroking directions were either spatially congruent (same direction of stroking motion on the participant's teeth and the dental model) or spatially incongruent (opposite directions: see Figure 3c). The order of the trials was randomised across the experiment for each participant. We performed nine trials in each condition. The methods otherwise resembled Experiment 1.

### 2.4 Experiments 4, 5 and 6: perceptual processes involved in the DMI

**Figure 4:**
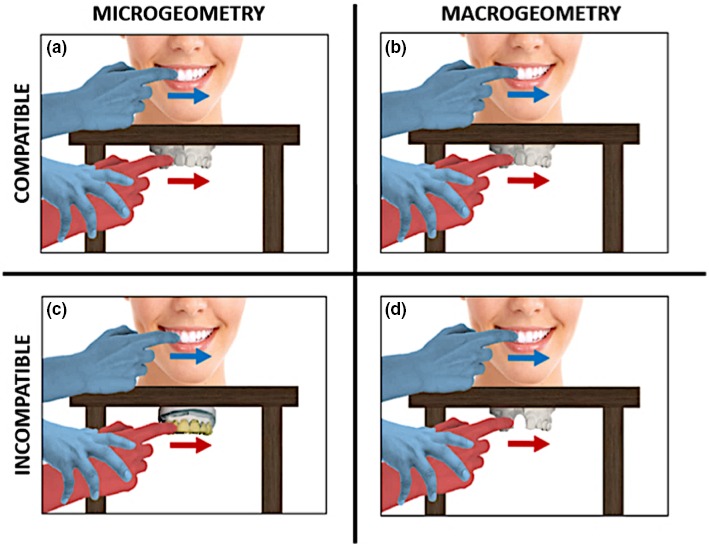
Variations of the compatibility between the somatosensory experiences of participant's teeth and those on the right index finger: Microgeometric and macrogeometric compatibility (a and b, Expts. 1, 2 and 3), microgeometric incompatibility (c, Expt. 4) and macrogeometric incompatibility (d, Expt. 5). [Colour figure can be viewed at http://www.wileyonlinelibrary.com]

In the second group of studies (Experiments 4, 5 and 6), the compatibility between the somatosensory experiences received on the participant's teeth and those received on their right index finger was modulated (see Figure 4). Microgeometric incompatibility (Experiment 4) and macrogeometric incompatibility (Experiment 5) were studied in order to investigate the relative importance of bottom‐up and top‐down processes for the DMI. To achieve this, the dental model that participants touched during the DMI paradigm was modified either in its microgeometric texture (Experiment 4) or in its macrogeometric structure (Experiment 5), to have morphological properties that differed from the participant's own teeth. In Experiment 4 (*N* = 8, 6 females, mean age ± SD: 21.4 ± 1.4 years), the texture of the dental model was modified across trials. In the incompatible microgeometric condition (10 trials), the facial surface of the central and lateral maxillary incisors of the dental model touched by participants was covered with ‘spiky’ Velcro (Sellotape^®^) (see Figure 2b). Therefore, the rough texture of the teeth that the participants felt with their right index fingers was inconsistent with the mechanical events generated by the experimenter stroking the participants’ own teeth, which had a much smoother surface. In the compatible microgeometric condition (10 trials), the dental model was as in Experiment 1 (see Figure 2a). Throughout the length of the whole experiment, participants wore headphones playing white noise to avoid the influence of acoustic clues to texture during the DMI paradigm. Other details of the method were as Experiment 1.

In Experiment 5 (*N* = 8, five females, mean age ± SD: 23.2 ± 2.3 years), the number of teeth in the dental model was manipulated across trials. In the incompatible macrogeometric condition (10 trials), the right maxillary incisor of the dental model was removed (see Figure 2c). In the compatible macrogeometric condition (10 trials), the dental model was intact (see Figure 2a). Other aspects of the methods were the same as in Experiment 1.

Experiment 6 (*N* = 8, four females, mean age ± SD: 26.4 ± 5.8 years) was a manipulation check to investigate whether participants could discriminate the presence of Velcro on a dental model with their right index finger, thus underscoring the potential relevance of Experiment 4. Each participant was invited to sit on a chair placed in front of a table. A total of 40 trials were tested. During each trial, participants were blindfolded and they were asked to wear headphones playing white noise, to avoid the use of any visual or acoustic clues to perform the task. In each trial, participant's right index finger was guided by the experimenter to touch the central and the lateral maxillary incisors of two different dental models placed on the table. Each touch lasted 2 s. After the stimulation period, the two dental models were removed from the table and the participant was instructed to remove the blindfold. Participants were then asked to judge whether the textures of the two dental models were the same or different, by pressing a button on a keyboard. Each model could have either a normal (see Figure 2a) or a modified texture (see Figure 2b) as in Experiment 4, with the models being manipulated independently. The order of trials was randomised for each participant.

### 2.5 Data analysis

On each trial, a pre‐test baseline judgement prior to stimulation and a post‐test judgement after stimulation were obtained for the maxillary incisors (Experiments 1–5), and for the tip of the nose (Experiments 1 and 3). We tested whether tactile stimulation of the DMI paradigm could elicit any change in the perceived position of the body parts aforementioned. We therefore calculated judgement errors as the difference between the real and the perceived position of the body parts of interest. Judgement errors were computed for both the pre‐test and the post‐test judgements. By *proprioceptive drift* (Tsakiris & Haggard, 2005), we refer to the difference between the post‐test and the pre‐test judgement errors, with positive drift indicating a drift towards the dental model. This measure has been widely used in the literature on bodily awareness to indicate the strength of multisensory illusions (Longo & Haggard, 2012; Tsakiris & Haggard, 2005). However, some studies have noted that this measure can dissociate from explicit reports, and have therefore recommend caution when using it (Abdulkarim & Ehrsson, 2016; Rohde, Luca, & Ernst, 2011).

## 3 RESULTS

### 3.1 Proprioceptive drift

**Figure 5:**
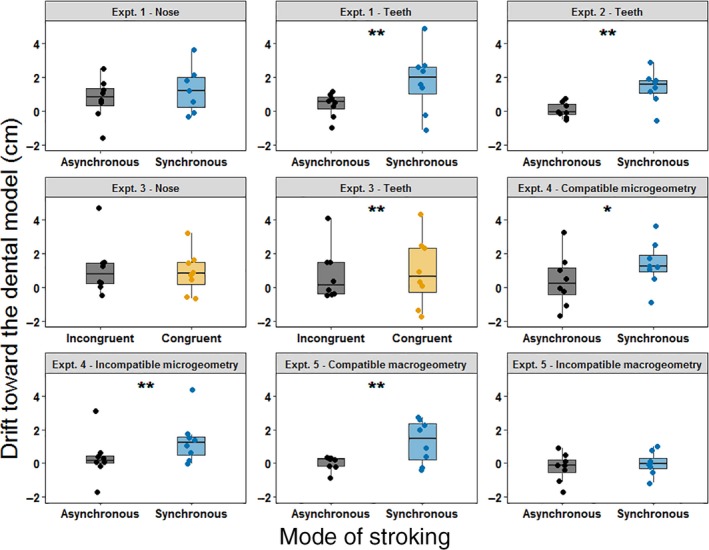
Box plots showing the proprioceptive drift across different stroking conditions in Experiments 1–5. Each single‐subject value corresponds to the proprioceptive drift towards the dental model (in cm). In all the box plots, lower and upper box boundaries represent 25th and 75th percentiles, respectively, and the line inside box shows the median. *p < .05, **p < .01. [Colour figure can be viewed at http://www.wileyonlinelibrary.com]

Several ANOVAs were computed on the proprioceptive drift in Experiments 1, 3, 4 and 5 Results of the ANOVAs are summarised in Table 1 and Figure 5. In Experiment 1, a 2 × 2 repeated‐measures ANOVA was computed to investigate the effect of spatio‐temporal tactile stimulation (synchronous vs. asynchronous) on the proprioceptive drift in different body parts (maxillary central incisors vs. nose). In Experiment 3, a 2 × 2 repeated‐measures ANOVA was performed to investigate the effect of spatial tactile stimulation (congruent vs. incongruent) on the proprioceptive drift in different body parts (maxillary central incisors vs. nose). Lastly, two further ANOVAs were computed to investigate the effect of microgeometry (Expt. 4, compatible vs. incompatible) and macrogeometry (Expt. 5, compatible vs. incompatible) on the proprioceptive drift across different stroking conditions (synchronous vs. asynchronous).

**Table 1:** Summary table reporting the results of the ANOVAs computed on the proprioceptive drift in Experiments 1, 3, 4 and 5. Significant results are presented in bold

	Proprioceptive drift (Experiments 1, 3, 4 and 5)				
	Source of variation	df	Effect size (η_p_)^2^	*F* Test	*p* level
Experiment 1 Spatio‐temporal pattern of stimulation	Stroking	1	0.387	4.417	.074
	Body part	1	0.112	0.881	.379
	**Stroking × Body part**	**1**	**0.526**	**7.758**	**.027**
Experiment 3 Spatial pattern of stimulation	Stroking	1	0.004	0.031	.866
	Body part	1	0.054	0.399	.548
	Stroking × Body part	1	0.187	1.615	.244
Experiment 4 Microgeometric modulations	**Stroking**	**1**	**0.878**	**50.341**	**<.001**
	Microgeometry	1	0.004	0.030	.868
	Stroking × Microgeometry	1	0.001	0.006	.941
Experiment 5 Macrogeometric modulations	**Stroking**	**1**	**0.593**	**10.181**	**.015**
	**Macrogeometry**	**1**	**0.785**	**25.523**	**.001**
	Stroking × Macrogeometry	1	0.351	3.788	.093

Experiment 1 showed a significant interaction between the stroking condition and the body part judged (see table). To further investigate this interaction, we used simple effects analysis (Howell, 1997) to compare the proprioceptive drift between synchronous and asynchronous conditions for each body part. Differences between synchronous and asynchronous conditions were significant only when participants were asked to estimate the position of their maxillary central incisors (*t*(7) = 2.609, *p* = .035, two‐tailed) and not when they were asked to estimate the position of their nose (*t*(7) = 0.404, *p* = .698, two‐tailed).

In Experiment 2, we observed a difference in the proprioceptive drift between synchronous and asynchronous stroking conditions (t(7) = 3.566, *p* = .009, two‐tailed). No significant interactions were observed in the ANOVAs computed in Experiments 3, 4 and 5.

In addition to classical frequentist statistics, Bayesian repeated‐measures ANOVAs and Bayesian paired samples t tests were also computed on the proprioceptive drift. Results of these analyses are reported as supporting information.

### 3.2 Sense of teeth ownership

**Figure 6:**
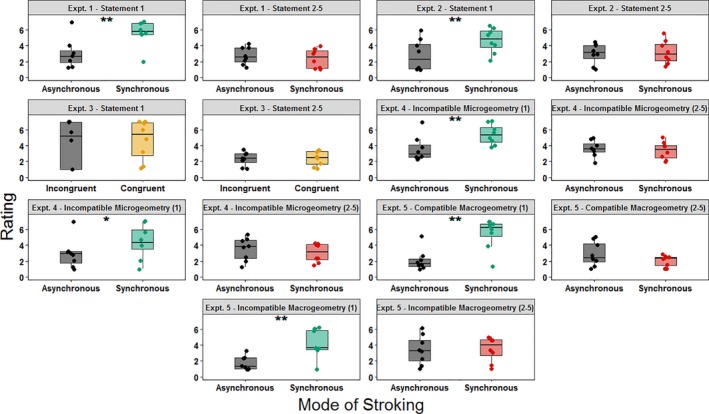
Box plots showing the rating to the statements of the dental model illusion Questionnaire obtained in Experiments 1–5. Each single‐subject value corresponds to the rating to the given DMI questionnaire's statement. In all the box plots, lower and upper box boundaries represent 25th and 75th percentiles, respectively, and the line inside box shows the median. *p < .05, **p < .01. [Colour figure can be viewed at http://www.wileyonlinelibrary.com]

All data were assessed for normality using the Shapiro–Wilk test (*p* > .05), and the appropriate non‐parametric tests were applied when one or more of the corresponding data sets failed to meet the criteria for normal distribution. More specifically, questionnaire data in Experiments 1, 3 and 5 were not normally distributed. In these cases, we ran both parametric and non‐parametric tests, and found similar patterns of significance. Parametric tests are more commonly used for factorial designs such as ours (Mircioiu & Atkinson, 2017), and we report the parametric tests in the paper, and non‐parametric tests for Experiments 1, 3 and 5 in supporting information. To analyse the effect of the DMI on the sense of ownership for teeth, we compared the rating expressed by participants on the first statement and on the averaged rating given for statements 2–5 of the DMI questionnaire between synchronous and asynchronous conditions. Figure 6 summarises the main results of the effect of the DMI on the sense of teeth ownership. In Experiment 1, this difference was significant for statement 1 (*t*(7) = 5.082, *p* = .001, two‐tailed), suggesting that participants were more likely to believe they were touching their own teeth after synchronous stimulation. No differences were found for the average of the other four statements (*t*(7) = −1.409, *p* = .201, two‐tailed).

In Experiment 2, the difference in rating between stroking conditions was significant for statement 1 (*t*(7) = 4.378, *p* = .003, two‐tailed), but not for the average of the other four statements (*t*(7) = 0.338, *p* = .745, two‐tailed).

In Experiment 3, questionnaire ratings did not show statistically significant differences across stroking conditions (all *p* values > .207). Consequently, neither the sense of teeth ownership nor participants’ suggestibility was modulated across different stroking conditions.

To analyse the results of the Dental Model Illusion questionnaire observed in Experiments 4 and 5, four 2 × 2 repeated‐measure ANOVAs were computed on the rating given to the first statements of the questionnaire and on the average rating expressed for statements 2–5. In Experiment 4, all the ANOVAs had as factors the microgeometry of the dental model used during each trial (compatible vs. incompatible) and the condition of stroking (synchronous vs. asynchronous). Results of the ANOVAs are summarised in table 2. In Experiment 5, all the ANOVAs had as factors the macrogeometry of the dental model used during each trial (compatible vs. incompatible) and the condition of stroking (synchronous vs. asynchronous). Results of the ANOVAs are summarised in Table 3.

**Table 2:** Summary table reporting the results of the ANOVAs computed on the rating given by participants to the first statement and to the average of statements 2–5 of the DMI questionnaire in Experiment 4. Significant results are presented in bold

	Experiment 4: Microgeometric modulations				
	Source of variation	df	Effect size (η_p_)^2^	*F* Test	*p* level
Statement 1 *‘I felt as if I was touching my teeth with my right index finger’*	**Stroking**	**1**	**0.668**	**14.114**	**.007**
	Microgeometry	1	0.375	4.197	.080
	Stroking × Microgeometry	1	0.063	0.468	.516
Statements 2–5 *Averaged rating*	Stroking	1	0.113	0.888	.377
	Microgeometry	1	0.049	0.363	.566
	Stroking × Microgeometry	1	0.045	0.326	.586

**Table 3:** Summary table reporting the results of the ANOVAs computed on the rating given by participants to the first statement and to the average of statements 2–5 of the DMI questionnaire in Experiment 5. Significant results are presented in bold

	Experiment 5: Macrogeometric modulations				
	Source of variation	df	Effect size (η_p_^2^)	F Test	*p* level
Statement 1 *‘I felt as if I was touching my teeth with my right index finger’*	**Stroking**	**1**	**0.815**	**30.903**	**.001**
	**Macrogeometry**	**1**	**0.513**	**7.365**	**.030**
	Stroking × Macrogeometry	1	0.328	3.424	.107
Statements 2–5 *Averaged rating*	Stroking	1	0.096	0.740	.418
	**Macrogeometry**	**1**	**0.658**	**13.462**	**.008**
	**Stroking × Macrogeometry**	**1**	**0.605**	**10.704**	**.014**

A simple effects analysis was conducted to further investigate the significant interaction observed in the ANOVA computed on the averaged rating of statements 2–5 in Experiment 5. Data showed that when the mode of stroking was synchronous, the averaged rating expressed to the control statements for suggestibility was higher when the dental model used during the DMI paradigm was macrogeometrically incompatible (*t*(7) = 4.325, *p* = .003, two‐tailed). That was not the case after asynchronous stimulation (*t*(7) = 1.728, *p* = .127, two‐tailed).

In addition to classical frequentist statistics, Bayesian repeated‐measures ANOVAs and Bayesian paired samples t tests were also on the rating for the DMI questionnaire's statements in Experiments 1–5. Results of these analyses are reported as supporting information.

### 3.3 Experiment 6: Velcro discrimination with the right index finger

In Experiment 6, we calculated the mean percentage of accuracy in the detection of the difference in texture of two dental models participants touched with their right index finger. The observed mean accuracy across our sample was 93.75% with a standard deviation of 11.288%. We performed a one sample *t* test to compare the observed accuracy with the accuracy that would be observed at chance level (50%). We concluded that the mean accuracy observed in our sample was statistically different from chance level (*t*(7) = 18.520, *p* < .001), suggesting that participants were able to detect the presence of Velcro when touching a dental model with their right index finger.

### 3.4 Overall effect size

**Figure 7:**
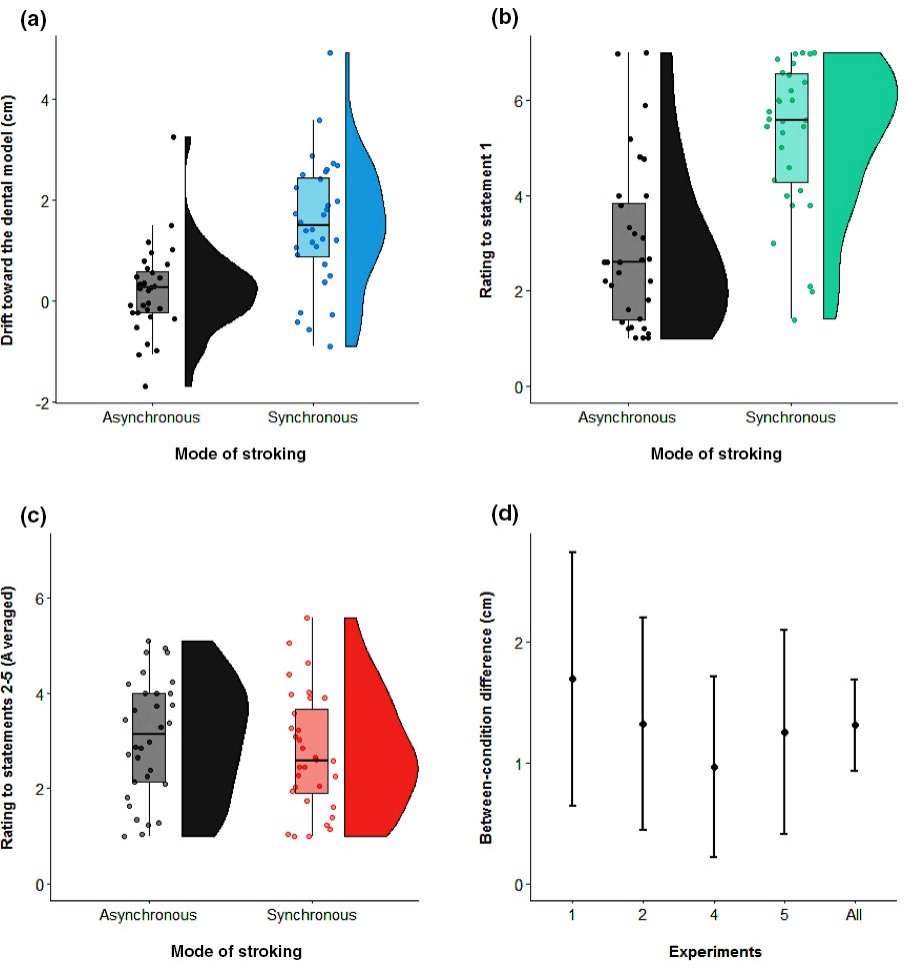
Raincloud plot (Allen, Poggiali, Whitaker, Marshall, & Kievit, 2018) of the pooled data across experiments (*N* = 32), for the two stroking conditions: asynchronous and synchronous. Each single‐subject value corresponds to the proprioceptive drift towards the dental model (in cm). Positive values correspond to a drift in the perceived position of the maxillary incisor towards the dental model. The half violin plot depicts the probability density of the data at different values and contains 95% confidence intervals of the mean for the two conditions (a). Raincloud plot of the pooled data across experiments (*N* = 32), for the two stroking conditions: asynchronous and synchronous. Each single‐subject value corresponds to the rating to the first statement of the DMI questionnaire. The half violin plot depicts the probability density of the data at different values and contains 95% confidence intervals of the mean for the two conditions (b). Raincloud plot of the pooled data across experiments (*N* = 32), for the two stroking conditions: asynchronous and synchronous. Each single‐subject value corresponds to the pooled rating of statements 2–5 of the DMI questionnaire. The half violin plot depicts the probability density of the data at different values and contains 95% confidence intervals of the mean for the two conditions (c). 95% confidence intervals of the difference between the two stroking conditions. Positive values correspond to a larger DMI effect for synchronous vs. asynchronous stroking condition. The differential DMI stroking effect is plotted for each experiment (*N* = 8), as well as the pooled data across experiments (*N* = 32). [Colour figure can be viewed at http://www.wileyonlinelibrary.com]

**Figure 8:**
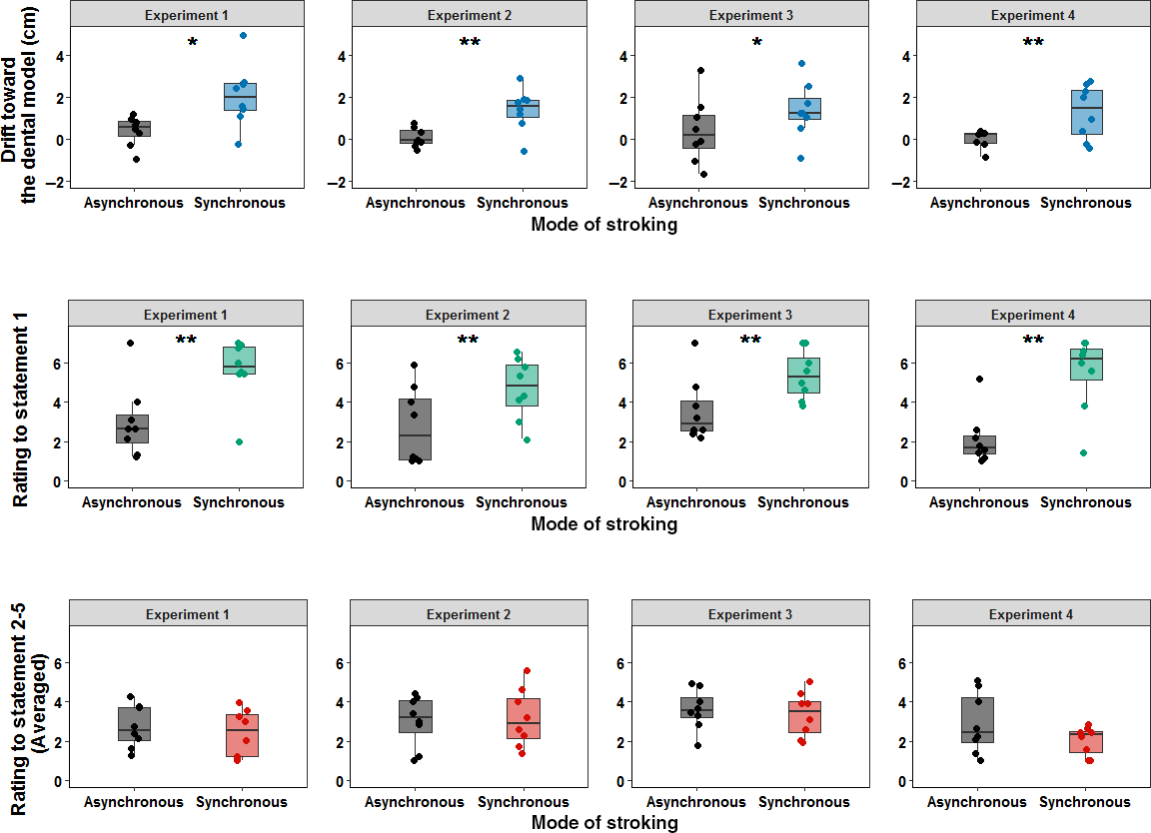
2 × 3 pooled ANOVAs computed with stroking as within‐subject factor and experiment as a between‐subject factor on the proprioceptive drift (a) on the rating given to the first statement of the DMI questionnaire (b) and on the pooled rating of statements 2–5 (c). Each single‐subject value corresponds to the proprioceptive drift towards the dental model (in cm) (a) and to the rating to the given DMI questionnaire's statement (b and c). In all the box plots, lower and upper box boundaries represent 25th and 75th percentiles, respectively, and the line inside box shows the median. *p < .05, **p < .01. [Colour figure can be viewed at http://www.wileyonlinelibrary.com]

To summarise the effect of mode of stroking on both the proprioceptive drift and the sense of teeth ownership, we also computed the magnitude of the DMI effect in Experiments 1, 2, 4 and 5 on the pooled data across experiments, taking the difference between synchronous and asynchronous condition drifts as an estimate of DMI effect size. In Figure 7, we report the confidence intervals and the probability density of the pooled data across experiments. Data are shown separately for the two key stroking conditions (synchronous and asynchronous) on the proprioceptive drift (Figure 7a), on the rating expressed to the first statement of the DMI questionnaire (Figure 7b) and on the averaged rating expressed to statements 2–5 (Figure 7C). Mean confidence intervals (CI) of this DMI effect for each experiment separately, as well as for the pooled data across experiments, are summarised in Figure 7d. Furthermore, several pooled ANOVAs were conducted across the 32 total participants in Experiments 1, 2, 4 and 5 (see Figure 8a). Data were pooled across experiments with factors of stroking (synchronous vs. asynchronous) and experiment number (Exp. 1, 2, 4 and 5, between subjects). Other, experiment‐specific factors were suppressed. The first ANOVA was computed on the proprioceptive drift. The main effect of stroking was statistically significant (*F*_1,28_ = 48.712, *p* < .001, *p* ^2^ = .635). Nor the main effect of experiment (*F*_3,28_ = 0.543, *p* = .657, *p* ^2^ = .055), neither the interaction of these two factors was statistically significant (*F*(3, 28) = 0.636, *p* = .598, *p* ^2^ = .064).

Finally, 2 further pooled ANOVAs were conducted. One was performed on the rating to the first statement of the questionnaire and the second one on the pooled rating to statements 2–5 across the same 32 total participants (see Figure 8b and c). In the ANOVA performed on the rating to the first statement, only the main effect of stroking (*F*_1,28_ = 87.549, *p* < .001, *p* ^2^ = .758) was statistically significant. Neither the main effect of experiment (*F*_3,28_ = 0.493, *p* = .690, *p* ^2^ = .050) nor the interaction of these two factors was statistically significant (*F*_3,28_ = 1.967, *p* = .142, *p* ^2^ = .174). On the other hand, in the ANOVA performed on the pooled ratings to statements 2–5, the main effect of stroking was not statistically significant (*F*_1,21_ = 2.062, *p* = .162, *p* ^2^ = .069). In addition, neither the main effect of experiment (*F*_3,28_ = 1.681, *p* = .194, *p* ^2^ = .153) nor the interaction of these two factors was statistically significant (*F*_3,28_ = 0.951, *p* = .430, *p* ^2^ = .092).

## 4 GENERAL DISCUSSION

We have performed the first experiments, to our knowledge, on the representation of one's teeth, using a modified version of a popular somatosensory illusion: the dental model illusion. In Experiment 1, we stimulated participant's teeth and a dental model either synchronously or asynchronously, and with or without spatial congruence. When participants touched a dental model and their own teeth were stroked at the same time (Experiment 1), we found an altered sense of body ownership in a qualitative questionnaire measure, and a shift in the perceived position of the participant's own teeth towards the location where they had touched the dental model. Experiment 2 showed that these same modulations in the proprioceptive drift and sense of teeth ownership were again observed when the overall amount of stimulation was exactly balanced between synchronous and asynchronous conditions. The temporal rather than the spatial aspect of the manipulations seemed most important, as no modulation of the sense of teeth ownership or teeth proprioception emerged in Experiment 3 in which we manipulated spatial congruence but not temporal synchrony (Experiment 3). Costantini and Haggard (2007) showed that spatial incongruency of up to 30° between the orientation of viewed and felt stroking movements did not abolish the RHI. Importantly, in their study the participant's hand was out of sight, so the incongruency may simply have been undetected. Our findings, in the case of teeth stimulation, again suggest that synchronised sensory inputs can cause illusions of bodily awareness even when the spatial features of the two sensory inputs differ. Altogether these findings suggest the DMI, like the somatic rubber hand illusion (Aimola Davies et al., 2013), is relatively robust to purely spatial incongruencies. Consequently, the mere synchronisation of tactile stimulation appears to be not only a necessary, but also a sufficient condition in order to produce changes in the spatial localisation and in the sense of ownership of teeth. This pattern of results suggests a dominant role of intersensory temporal synchrony in this, as in other manipulations of ownership (Tsakiris & Haggard, 2005). However, caution is required in interpreting these data as the Bayesian ANOVAs conducted in Experiment 3 provide only moderate evidence in support of the null hypothesis, namely that proprioceptive drift and subjective rating to statement 1 of the DMI questionnaire are independent of purely spatial modulation of the stroking stimulation.

We found clear body part specificity in the dental model illusion. Synchronous self‐touch caused a displacement in the perceived position of the teeth, but not in the perceived position of the nose. These effects amounted to a local, tooth‐specific proprioceptive drift. During our DMI paradigm, participants believed they were touching their own teeth. However, the dental model they actually touched was located 8 cm below their actual teeth. Consequently, during the DMI, people experience a downward shift in the spatial position of their own teeth. In many studies of bodily awareness, altered representation of a single body segment induces a more general update of body representation, in order to preserve its overall coherence (Lackner, 1988). For example, the ‘Phantom Nose Illusion’ (Ramachandran & Hirstein, 1998) depends on the correlation between two tactile events. One touch is delivered by the experimenter's finger on the participant's nose. The other touch consists of the participant's finger touching the nose of another subject placed just in front of them. As the two touches are synchronous, the participant experiences a subjective sensation of nose elongation. We did not find evidence for this kind of coherence‐preserving transfer between linked body parts in the DMI. Rather, a modular organisation (Haggard & Wolpert, 2005) was found, in which local distortions in body representation might be inconsistent with representation of nearby body parts. For example, item 3 of the DMI questionnaire specifically investigated whether DMI could induce a perceived elongation of teeth by asking participants whether they perceived their teeth to be modified in their size after tactile illusion. In principle, the different spatial locations of the afferent information from the participant's teeth and from their fingertip could be reconciled if the teeth were perceived as longer than their true length (de Vignemont, Ehrsson, & Haggard, 2005). As Experiments 1–5 show, the average rating given to this statement did not differ between synchronous and asynchronous conditions. Consequently, the perceived position of the teeth was influenced by DMI, but the size of the teeth was not. This result suggests that local inconsistencies are tolerated when multisensory stimulation drives plastic adaptations of body representation. The perceived position of the upper incisors changed, without any comparable change in the perceived position of the nose—despite the fixed anatomical relation between these two body parts. Interestingly, the receptors that underlie mechanosensitivity of the teeth are located on periodontal ligaments, which attach the root of the tooth to the alveolar bone (Trulsson & Johansson, 1996, 2002). Thus, the perceived position of the teeth is directly linked, at receptor level, to the perceived position of the upper jaw itself. However, the perceived position of the nose appeared to be handled separately.

Tactile illusions have been used widely in neuroscientific literature to study the processes behind multisensory integration and bodily awareness. The interplay between vision, touch and proprioception has have been a key focus of interest (Botvinick & Cohen, 1998; Ehrsson et al., 2005; Tsakiris & Haggard, 2005). Scientific evidence is divided over whether correlation between multisensory inputs is sufficient for attribution of body parts. On the one hand, Armel and Ramachandran (2003) argue that the RHI illusion is the result of a mere bottom‐up perceptual learning. According to their view, the combination of time‐correlated sensory inputs is a sufficient condition to update proprioception and body ownership. For example, they found that people could develop a weak RHI effect when a tabletop was stroked in synchrony with their own hand. However, caution is required in interpreting these data as other studies did not find any reliable illusion for non‐body objects (Tsakiris, Carpenter, James, & Fotopoulou, 2010). In fact, Tsakiris and Haggard (2005) found stronger proprioceptive drifts when viewing synchronous stroking of a rubber hand, compared to an inanimate object. They argued that a ‘top‐down’ representation, that the stroked object was plausibly hand‐like, was relevant to the RHI. In the same way, our dental model was plausibly like the participants’ own dentition. However, the DMI differs from the RHI in two main respects. Firstly, it does not involve visual inputs, which dominate touch and proprioception in many multisensory paradigms, not only the RHI (Ernst & Banks, 2002). Secondly, the DMI involves sensory inputs to body parts that are cannot move independently. The maxillary incisors are attached to the skull via the jaw. They may thus have a fixed position relative to the putative human *egocentre* (Barbeito & Ono, 1979). The role of top‐down information in bodily illusions for such fixed body parts has not previously been studied, to our knowledge. Experiments 4 and 5 aimed to explore the influence of top‐down and bottom‐up processes in the DMI. In order to study these effects, the microgeometric compatibility of the tooth surface (Experiment 4) and the macrogeometric compatibility of the dentition morphology (Experiment 5) in the plaster model were altered, to induce conflict between the somatosensory experiences arising on the participant's own teeth and from their right index finger's stroking of the dental teeth model.

In particular, in Experiment 4 we compared a synchronous pattern and asynchronous pattern of stimulation using two different types of dental models. In one, the texture of central and lateral incisors was modified by covering them with rigid Velcro. The texture of the other was unmodified and equal to the one used in Experiment 1, 2 and 3. A control experiment (Experiment 6) demonstrated that this texture modification was perceptually evident to participants. Explicit and implicit (proprioceptive) measures showed no differences between these two microgeometric experiences. Bayesian analysis confirmed that the best model of the proprioceptive drift did not include a main effect of microgeometry. On the other hand, microgeometry is included in the most explanatory model of teeth ownership. However, this analysis provides moderate evidence against the exclusion of microgeometry in a model of proprioception and teeth ownership. We conclude that surface texture (Yoshioka & Zhou, 2009) is not a major modulator of tooth ownership. These findings are in line with the RHI literature, which shows that RHI does not depend on conceptual interpretation of tactile sensations (Schütz‐Bosbach, Tausche, & Weiss, 2009). People commonly sample the surface texture of the teeth with the tongue, particularly after eating, drinking or sleeping. However, our results indicate that these textural aspects exert only a minimal influence on the representation of the teeth themselves. We speculate that microgeometric textural modulations are attributed to external stimuli, such as food particles, that adhere to the tooth surface, rather than to one's own body.

Macrogeometric haptic perception of object shape requires extensive somatosensory processing, subsequent to simple somatosensory encoding (O'Sullivan, Roland, & Kawashima,^1994^). Therefore, Experiment 5 sought to investigate the effects of macrogeometric similarity on the DMI. We replicated the trend from Experiments 1, 2 and 4, with stronger proprioceptive drift in synchronous conditions. Interestingly, the changes due to synchronous stroking showed a tendency to depend on the structure of the dental model. When the dental model used in the paradigm had a missing incisor, proprioception (but not the explicit judgements of teeth ownership) showed a trend towards a reduced DMI. Bayesian analysis showed that macrogeometry played a role in explaining both proprioceptive drift and explicit judgements of teeth ownership. This suggests some top‐down influence of a cognitive dental model that includes macrogeometric features. The comparison of an internal model of our participants’ complete dentition and the finger's tactile experience of a tooth missing from the model would produce a conflict, so that what is felt on the teeth and with the finger no longer matches. The interaction between macrogeometry and synchrony achieved only trend‐level significance, so caution is required in interpreting this result, particularly given the small number of participants. On the other hand, the direction of the interaction could be clearly predicted by previous studies of RHI. Lastly, no differences in rating emerged between stroking conditions for the average of the control statements for suggestibility. In conclusion, we find a modest evidence for a top‐down, macrogeometric component of the DMI. We found no convincing evidence that a prior representation of tooth surface texture contributes to the sense of ownership with respect to one's own teeth. Conversely, we found moderate evidence that a macrogeometric model of dentition morphology could contribute to the sense of one's own teeth. We plan to replicate this latter result in further studies. Many people remember the strange and vivid experience of exploring with their tongue the gap left after a tooth falls out or is removed. Our results suggest interesting opportunities for longitudinal studies on updating internal models of one's own body before and after tooth loss.

The relative positions of some body parts are fixed (e.g. nose and eyes), while the relative positions of other body parts vary frequently (e.g. hands and eyes). The head is often considered as the stable centroid of bodily experience (Alsmith & Longo, 2014). The experiments reported in this article challenge this model by showing that the perceived position of teeth is not stable but is subject to rapid plastic change through experience.

The position of the mouth in space is thus plastic, experience‐dependent and continuously updated based on sensory inputs. The hand‐to‐mouth movement is critically important for feeding, and thus for survival. It has long been considered a motor primitive (Graziano, 2006; Rizzolatti & Arbib, 1998) and an innate motor pattern (Butterworth & Hopkins, 1988). For example, these movements occur in utero, as early as the 14th–22nd weeks of gestational period (Zoia et al., 2013). However, moving the hand to the mouth presupposes a representation of where the mouth is in space. Nativist theories presumably consider this a privileged spatial datum, which may be innately specified. Our research casts doubt on such views, as we show that the perceived position of the mouth is readily modified by current sensorimotor experiences. We speculate that infants, and perhaps foetuses, may learn the position of their own mouth, and build a hand‐to‐mouth body model, by trial‐and‐error (Farah, 1998; Schillaci & Hafner, 2011).

On this view, the DMI might simply reflect Bayesian integration between the signals generated on the two sensory surfaces (the teeth, stimulated by the experimenter) and the finger (stimulated by its movement across the dental model). In RHI, the proprioceptive drift is interpreted in terms of the high weighting given to vision of hand position. In DMI, by analogy, the drift towards the model would indicate a high weighting for the information generated by the haptic exploration of the model by the participant's guided finger movement. We speculate that experience of feeling the dental model as part of one's own body arises as a result of adaptation of teeth position information by finger position information. Where might this adaptation occur? The secondary somatosensory cortices seem to maintain an online representation of the body (Press, Heyes, Haggard, & Eimer, 2008; Tsakiris, Hesse, Boy, Haggard, & Fink, 2007a; Tsakiris, Schütz‐Bosbach, & Gallagher, 2007b) and are thus a plausible candidate. Posterior parietal cortices might also contribute. In fact, activity in these regions correlates with proprioceptive drift observed after RHI (Brozzoli, Gentile, & Ehrsson, 2012). This area also plays a critical role in representing spatial relations between body parts (Buxbaum & Coslett, 2001). Lastly, premotor cortices could potentially be involved in DMI. Indeed, the strength of self‐touch illusions has been shown to correlate with the activity in these regions (Ehrsson et al., 2005).

Somatosensory impairments occur in about half of stroke cases. Autotopagnosia is a disorder of the body schema characterised by an inability to localise and orient different parts of the body in space (Buxbaum & Coslett, 2001; Felician, Ceccaldi, Didic, Thinus‐Blanc, & Poncet, 2003; Gainotti, Caltagirone, Carecchi, & Ibba, 1976). In particular, information about the relative position of one body part with respect to another seems to be lost. The representation of mouth position, and the ability to bring the hand to the mouth, is essential for feeding, and thus for independent living. Difficulties with independent feeding activities are often attributed to motor weakness, in both paediatric (Van den Engel‐Hoek, de Groot, de Swart, & Erasmus, 2015) and geriatric populations (Ahmed & Haboubi, 2010). However, we speculate that poor spatial representation of mouth position may also play a role. Self‐feeding behaviour is one of the first skills to deteriorate in Alzheimer's‐type dementia (LeClerc, Wells, Leclerc, & Wells, 1998). Feeding apraxia is common in advanced stages of the disease and, as a result, patients are unable to self‐feed. Assisted feeding and tube feeding are the most common solutions adopted in these cases (Hanson, Ersek, Gilliam, & Carey, 2011). A fairly recent study has shown that self‐touch can modulate impairments in the localisation of body parts in space while enhancing the sense of body ownership (Van Stralen, van Zandvoort, & Dijkerman, 2011). We have shown that humans can continuously update the position of their mouth in space through tactile inputs. This principle might be used as a core feature of a putative novel neuropsychological treatment to reinstate the representation of the mouth and of its spatial relations with other body parts. We speculate that patients exposed to multisensory, multi‐effector oral stimulation might show an enhancement in functional hand to mouth movements. This might potentially enhance oral proprioception and ownership, with consequent improvements in self‐feeding, and thus in quality of life.

## CONFLICTS OF INTEREST

Nothing to report.

## AUTHOR CONTRIBUTIONS

D.B. conceived the study; D.B. and P.H. designed the study; D.B. acquired and analysed data, and drafted figures; D.B. and P.H. wrote the paper.

## Data Availability

The authors confirm that the data supporting the findings of this study are available within the article and its supporting information.
