# Supplementary material for: Where is my mouth? Rapid experience‐dependent plasticity of perceived mouth position in humans
Source: Eur J Neurosci. 2019 Jul 18;50(11):3814–30. doi: 10.1111/ejn.14508 (PMC6973246; doi:10.1111/ejn.14508)
Supplement: Supplementary file 1 [file EJN-50-3814-s001.docx]

**Non-parametric tests**

Questionnaire data in experiments 1,3 and 5 were not normally distributed. A Wilcoxon signed-rank test was used to analyse pairwise comparisons in experiments 1 and 3. In experiment 5, the Friedman test was also used to compare multiple conditions. All the tests were two-tailed. In experiment 1 we observed a significant difference in the rating given by participants to the first statement of the DMI questionnaire across stroking conditions (Wilcoxon signed-rank test: Z = -2.414, p = .016). No such difference was observed across stroking conditions for the averaged rating given to statements 2-5 (Wilcoxon signed-rank test: Z = -1.134, p = .257).

In experiment 3 no differences were observed in rating given to statement 1 (Wilcoxon signed-rank test: Z = -1.000, p = .317) and to the average of the other four statements (Wilcoxon signed-rank test: Z = -.577, p = .564) across stroking conditions.

In experiment 5 ownership scores given to statement 1 (Friedman test; χ2 [3, n = 8] = 19.381, p < .001) and to statements 2-5 (Friedman test; χ2 [3, n = 8] = 9.980, p = .019) differed significantly across the four conditions. To test whether manipulating the macrogeometry of the dental model produced any difference in the rating given to the statements of the DMI questionnaire, we compared ownership scores between compatible (synchronous compatible and asynchronous compatible) and corresponding incompatible macrogeometry conditions (synchronous incompatible and asynchronous incompatible). For statement 1 ownership ratings did not differ between asynchronous incompatible and corresponding compatible condition (Z = -1.633, p=.102). This difference was only marginally statistically significant between synchronous compatible and synchronous incompatible conditions (Z = -2.032, p=.042). For statements 2-5, ownership ratings did not differ between asynchronous compatible and corresponding incompatible condition (Z = -1.000, p=.317). However, this difference was statistically significant between synchronous compatible and incompatible conditions (Z = -2.220, p=.026).

We then investigated whether manipulating the stroking factor produced the expected effect on ownership (i.e., higher ownership scores after synchronous stimulation). Therefore, we compared synchronous (compatible synchronous and incompatible synchronous) with the corresponding asynchronous conditions (compatible asynchronous and incompatible asynchronous). For statement 1 ownership ratings were higher in both synchronous conditions than in the corresponding asynchronous conditions (compatible synchronous versus compatible asynchronous: Z = -2.392, p = .017; incompatible synchronous versus incompatible asynchronous: Z = -2.388, p = .017). For statements 2-5, ownership ratings did not differ between all synchronous conditions and their corresponding asynchronous conditions (all p>.102).

**Dental Model Illusion Questionnaire Statements**

| Statement 1 | I felt as if I was touching my teeth with my right index finger. |
| --- | --- |
| Statement 2 | I felt as if I had more than one set of teeth. |
| Statement 3 | I felt as if my teeth were larger than normal. |
| Statement 4 | I felt as if my teeth had a different position. |
| Statement 5 | I felt as if I was unable to feel my own teeth |

**Bayes Factor Analysis**

Bayesian repeated measures ANOVAs and Bayesian paired samples t-tests have been computed with JASP. Bayesian repeated measures ANOVA generates several models to test the likelihood that the dependent variable (either proprioceptive drift or subjective ratings from the DMI questionnaire) depends on: anything (Model 1), one single main factor (Models 2 and 3) the combination of both main factors (Model 4) and the interaction between the two main factors (Model 5). The column "p(M)" shows the prior model probabilities, the column "p(M|data)" shows the posterior model probabilities, and the column "BF_M_" shows the change from prior to posterior model odds. The column "BF_10_" lists the Bayes factor for each model against the null model. The prior inclusion probability "P(incl)" is the sum of the prior probabilities of all models that include the effect. Similarly, the posterior inclusion probability "P(incl|data)" is the sum of the posterior probabilities of all models that include the effect. The inclusion Bayes factor "BF_Inclusion_" is the change from prior to posterior inclusion odds.

**Proprioceptive Drift**

**Experiment1: Model Comparison**

| **Models** | | **P(M)** | | **P(M\|data)** | | **BF _M_** | | **BF _10_** | |  |
| --- | --- | --- | --- | --- | --- | --- | --- | --- | --- | --- |
| 1. Null model |  | 0.200 |  | 0.152 |  | 0.717 |  | 1.000 |  |  |
| 1. Body Part |  | 0.200 |  | 0.063 |  | 0.268 |  | 0.414 |  |  |
| 1. Stroking |  | 0.200 |  | 0.349 |  | 2.148 |  | 2.299 |  |  |
| 1. Body Part + Stroking |  | 0.200 |  | 0.158 |  | 0.750 |  | 1.039 |  |  |
| 1. Body Part + Stroking +   Body Part ✻ Stroking |  | 0.200 |  | 0.278 |  | 1.540 |  | 1.829 |  |  |

| **Experiment 1: Analysis of Effects** | | | | | | | |
| --- | --- | --- | --- | --- | --- | --- | --- |
| **Effects** | | **P(incl)** | | **P(incl\|data)** | | **BF _Inclusion_** | |
| Body part |  | 0.600 |  | 0.506 |  | 0.684 |  |
| Stroking |  | 0.600 |  | 0.783 |  | 2.402 |  |
| Body Part ✻ Stroking |  | 0.200 |  | 0.280 |  | 1.559 |  |
|  | | | | | | | |

**Experiment 2**

|  | |  | |  | | **BF₁₀** |  |  |
| --- | --- | --- | --- | --- | --- | --- | --- | --- |
| Asynchronous |  | - |  | Synchronous |  | 7.008 |  |  |

**Experiment3: Model Comparison**

| **Models** | | **P(M)** | **P(M\|data)** | | | **BF _M_** | | **BF _10_** | |  |
| --- | --- | --- | --- | --- | --- | --- | --- | --- | --- | --- |
| 1. Null model |  | 0.200 |  | 0.503 |  | | 4.043 |  | 1.000 |  |
| 1. Body Part |  | 0.200 |  | 0.217 |  | | 1.108 |  | 0.431 |  |
| 1. Stroking |  | 0.200 |  | 0.168 |  | | 0.808 |  | 0.334 |  |
| 1. Body Part + Stroking |  | 0.200 |  | 0.072 |  | | 0.309 |  | 0.143 |  |
| 1. Body Part + Stroking +   Body Part ✻ Stroking |  | 0.200 |  | 0.041 |  | | 0.169 |  | 0.081 |  |

| **Experiment 3: Analysis of Effects** | | | | | | | |
| --- | --- | --- | --- | --- | --- | --- | --- |
| **Effects** | | **P(incl)** | | **P(incl\|data)** | | **BF _Inclusion_** | |
| Body part |  | 0.600 |  | 0.344 |  | 0.349 |  |
| Stroking |  | 0.600 |  | 0.268 |  | 0.244 |  |
| Body Part ✻ Stroking |  | 0.200 |  | 0.038 |  | 0.158 |  |
|  | | | | | | | |

**Experiment4: Model Comparison**

| **Models** | | **P(M)** | | **P(M\|data)** | | **BF _M_** | | **BF _10_** | |  |
| --- | --- | --- | --- | --- | --- | --- | --- | --- | --- | --- |
| 1. Null model |  | 0.200 |  | 0.005 |  | 0.021 |  | 1.000 |  |  |
| 1. Stroking |  | 0.200 |  | 0.670 |  | 8.131 |  | 125.738 |  |  |
| 1. Microgeometry |  | 0.200 |  | 0.002 |  | 0.007 |  | 0.332 |  |  |
| 1. Stroking + Microgeometry |  | 0.200 |  | 0.226 |  | 1.168 |  | 42.395 |  |  |
| 1. Stroking + Microgeometry +   Stroking ✻ Microgeometry |  | 0.200 |  | 0.097 |  | 0.428 |  | 18.127 |  |  |

| **Experiment 4: Analysis of Effects** | | | | | | | |
| --- | --- | --- | --- | --- | --- | --- | --- |
| **Effects** | | **P(incl)** | | **P(incl\|data)** | | **BF _Inclusion_** | |
| Microgeometry |  | 0.600 |  | 0.317 |  | 0.309 |  |
| Stroking |  | 0.600 |  | 0.993 |  | 95.551 |  |
| Microgeometry ✻ Stroking |  | 0.200 |  | 0.087 |  | 0.382 |  |
|  | | | | | | | |

**Experiment5: Model Comparison**

| **Models** | | **P(M)** | | **P(M\|data)** | | **BF _M_** | | **BF _10_** | |  |
| --- | --- | --- | --- | --- | --- | --- | --- | --- | --- | --- |
| 1. Null model |  | 0.200 |  | 0.006 |  | 0.025 |  | 1.000 |  |  |
| 1. Macrogeometry |  | 0.200 |  | 0.033 |  | 0.136 |  | 5.280 |  |  |
| 1. Stroking |  | 0.200 |  | 0.026 |  | 0.106 |  | 4.171 |  |  |
| 1. Macrogeometry + Stroking |  | 0.200 |  | 0.288 |  | 1.616 |  | 46.313 |  |  |
| 1. Macrogeometry + Stroking +   Macrogeometry ✻ Stroking |  | 0.200 |  | 0.647 |  | 7.344 |  | 104.213 |  |  |

| **Experiment 5: Analysis of Effects** | | | | | | | |
| --- | --- | --- | --- | --- | --- | --- | --- |
| **Effects** | | **P(incl)** | | **P(incl\|data)** | | **BF _Inclusion_** | |
| Macrogeometry |  | 0.600 |  | 0.967 |  | 19.376 |  |
| Stroking |  | 0.600 |  | 0.959 |  | 15.600 |  |
| Macrogeometry ✻ Stroking |  | 0.200 |  | 0.633 |  | 6.908 |  |
|  | | | | | | | |

**Teeth Ownership**

**Experiment 1: Model Comparison**

|  | |  | |  | | **BF₁₀** | |  |
| --- | --- | --- | --- | --- | --- | --- | --- | --- |
| Statement 1 |  |  |  | Asynchronous – Synchronous |  | 30.899 |  |  |
| Statement 2-5 (averaged) |  |  |  | Asynchronous – Synchronous |  | 0.706 |  |  |

**Experiment 2: Model Comparison**

|  | |  | |  | | **BF₁₀** | |  |
| --- | --- | --- | --- | --- | --- | --- | --- | --- |
| Statement 1 |  |  |  | Asynchronous – Synchronous |  | 15.970 |  |  |
| Statement 2-5 (averaged) |  |  |  | Asynchronous – Synchronous |  | 0.353 |  |  |

**Experiment 3: Model Comparison**

|  | |  | |  | | **BF₁₀** | |
| --- | --- | --- | --- | --- | --- | --- | --- |
| Statement 1 |  |  |  | Asynchronous – Synchronous |  | 0.693 |  |
| Statement 2-5 (averaged) |  |  |  | Asynchronous – Synchronous |  | 0.354 |  |

**Experiment 4: Model Comparison, Statement 1**

| **Models** | | **P(M)** | | **P(M\|data)** | | **BF _M_** | | **BF _10_** | |
| --- | --- | --- | --- | --- | --- | --- | --- | --- | --- |
| 1. Null model |  | 0.200 |  | 0.004 |  | 0.015 |  | 1.000 |  |
| 1. Microgeometry |  | 0.200 |  | 0.003 |  | 0.014 |  | 0.940 |  |
| 1. Stroking |  | 0.200 |  | 0.260 |  | 1.409 |  | 70.488 |  |
| 1. Microgeometry + Stroking |  | 0.200 |  | 0.497 |  | 3.945 |  | 134.366 |  |
| 1. Microgeometry + Stroking +   Microgeometry ✻ Stroking |  | 0.200 |  | 0.236 |  | 1.234 |  | 63.807 |  |

| **Experiment4: Analysis of Effects, Statements 1** | | | | | | | |
| --- | --- | --- | --- | --- | --- | --- | --- |
| **Effects** | | **P(incl)** | | **P(incl\|data)** | | **BF _Inclusion_** | |
| Microgeometry |  | 0.600 |  | 0.730 |  | 1.804 |  |
| Stroking |  | 0.600 |  | 0.993 |  | 91.902 |  |
| Microgeometry ✻ Stroking |  | 0.200 |  | 0.226 |  | 1.168 |  |
|  | | | | | | | |

**Experiment 4: Model Comparison, Statements 2-5 (averaged)**

| **Models** | | **P(M)** | | **P(M\|data)** | | **BF _M_** | | **BF _10_** | |  |
| --- | --- | --- | --- | --- | --- | --- | --- | --- | --- | --- |
| 1. Null model |  | 0.200 |  | 0.436 |  | 3.092 |  | 1.000 |  |  |
| 1. Microgeometry |  | 0.200 |  | 0.178 |  | 0.864 |  | 0.408 |  |  |
| 1. Stroking |  | 0.200 |  | 0.250 |  | 1.336 |  | 0.574 |  |  |
| 1. Microgeometry + Stroking |  | 0.200 |  | 0.094 |  | 0.415 |  | 0.216 |  |  |
| 1. Microgeometry + Stroking +   Microgeometry ✻ Stroking |  | 0.200 |  | 0.042 |  | 0.175 |  | 0.096 |  |  |

| **Experiment 4: Analysis of Effects - Statements 2-5 (averaged)** | | | | | | | |
| --- | --- | --- | --- | --- | --- | --- | --- |
| **Effects** | | **P(incl)** | | **P(incl\|data)** | | **BF Inclusion** | |
| Stroking |  | 0.600 |  | 0.387 |  | 0.421 |  |
| Microgeometry |  | 0.600 |  | 0.316 |  | 0.308 |  |
| Stroking ✻ Microgeometry |  | 0.200 |  | 0.043 |  | 0.181 |  |
|  | | | | | | | |

**Experiment 5: Model Comparison, Statement 1**

| **Models** | | **P(M)** | | **P(M\|data)** | | **BF _M_** | | **BF _10_** | |  |
| --- | --- | --- | --- | --- | --- | --- | --- | --- | --- | --- |
| 1. Null model |  | 0.200 |  | 4.960e -6 |  | 1.984e -5 |  | 1.000 |  |  |
| 1. Macrogeometry |  | 0.200 |  | 3.176e -6 |  | 1.271e -5 |  | 0.640 |  |  |
| 1. Stroking |  | 0.200 |  | 0.227 |  | 1.178 |  | 45859.739 |  |  |
| 1. Macrogeometry + Stroking |  | 0.200 |  | 0.452 |  | 3.293 |  | 91027.275 |  |  |
| 1. Macrogeometry + Stroking +   Macrogeometry ✻ Stroking |  | 0.200 |  | 0.321 |  | 1.891 |  | 64707.160 |  |  |

| **Experiment 5: Analysis of Effects – Statement 1** | | | | | | | |
| --- | --- | --- | --- | --- | --- | --- | --- |
| **Effects** | | **P(incl)** | | **P(incl\|data)** | | **BF Inclusion** | |
| Stroking |  | 0.600 |  | 1.000 |  | 89812.800 |  |
| Macrogeometry |  | 0.600 |  | 0.753 |  | 2.034 |  |
| Stroking ✻ Macrogeometry |  | 0.200 |  | 0.289 |  | 1.626 |  |
|  | | | | | | | |

**Experiment 5: Model Comparison, Statements 2-5 (averaged)**

| **Models** | | **P(M)** | | **P(M\|data)** | | **BF _M_** | | **BF _10_** | |  |
| --- | --- | --- | --- | --- | --- | --- | --- | --- | --- | --- |
| 1. Null model |  | 0.200 |  | 0.066 |  | 0.282 |  | 1.000 |  |  |
| 1. Macrogeometry |  | 0.200 |  | 0.430 |  | 3.016 |  | 6.537 |  |  |
| 1. Stroking |  | 0.200 |  | 0.032 |  | 0.134 |  | 0.494 |  |  |
| 1. Macrogeometry + Stroking |  | 0.200 |  | 0.235 |  | 1.226 |  | 3.567 |  |  |
| 1. Macrogeometry + Stroking +   Macrogeometry ✻ Stroking |  | 0.200 |  | 0.237 |  | 1.244 |  | 3.608 |  |  |

| **Experiment 5: Analysis of Effects - Statements 2-5 (averaged)** | | | | | | | |
| --- | --- | --- | --- | --- | --- | --- | --- |
| **Effects** | | **P(incl)** | | **P(incl\|data)** | | **BF Inclusion** | |
| Stroking |  | 0.600 |  | 0.504 |  | 0.676 |  |
| Macrogeometry |  | 0.600 |  | 0.902 |  | 6.118 |  |
| Stroking ✻ Macrogeometry |  | 0.200 |  | 0.235 |  | 1.225 |  |
|  | | | | | | | |
