# Supplementary material for: Where is my mouth? Rapid experience‐dependent plasticity of perceived mouth position in humans
Source: Eur J Neurosci. 2019 Jul 18;50(11):3814–30. doi: 10.1111/ejn.14508 (PMC6973246; doi:10.1111/ejn.14508)
Supplement: Supplementary file 2 [file EJN-50-3814-s002.pdf]

EJN European Journal of Neuroscience FENS Federation of European Neuroscience Societies

Where is my mouth? Rapid experience-dependent plasticity of perceived mouth position in humans

Bono, Davide (contact); Haggard, Patrick

Review timeline:

Submission date: 30-Nov-2018
Editorial Decision: Minor Revision
Revision Received: 04-Mar-2019
Editorial Decision: Minor Revision
Revision Received: 15-May-2019
Accepted: 01-Jul-2019

Editor: Sophie Molholm
Reviewer 1: Lorenzo Pia
Reviewer 2: Peter Brugger
Reviewer 3: Henrik Ehrsson

1st Editorial Decision 30-Jan-2019

Dear Mr. Bono,

Three reviewers and one of our section editors have now evaluated your manuscript. In general they find the approach to be appropriate, and the findings of potential interest to the EJN readership. The reviewers raise some issues/questions that you will need to address in your response letter, and in the manuscript as appropriate. We look forward to receiving your response and revised manuscript.

Please provide a graphic abstract and text, describe whether consent was written or verbal and remove any bullet points from the text.

When revising the manuscript, please embolden or underline major changes to the text so they are easily identifiable and DO NOT leave 'track change' formatting marks in your paper. Please ensure that you provide a text and a figure file for the Graphical Abstract (as detailed in the instructions below). When carrying out your revisions please refer to the checklist below and visit the EJN author guidelines at www.ejneuroscience.org

When finalized, please upload your complete revised manuscript onto the website, as a Word file (.doc, or .docx). Please also ensure that a complete set of tables and figures is included as separate files, even if these have not changed from the originals. At this stage it is necessary to provide high resolution figures. Please see important instructions below.

Please go into https://mc.manuscriptcentral.com/ejn - Author Centre - manuscripts with decisions where you will find a 'create a revision' link under 'actions'. We ask that you please indicate the way in which you have responded to the points raised by the Editors and Reviewers in a letter. Please upload this response letter as a separate Word (.doc or PDF) file using the file designation "Authors' Response to Reviewers" when uploading your manuscript files. Please DO NOT submit your revised manuscript as a new one. Also, please note that only the Author who submitted the original version of the manuscript should submit a revised version.

If you are able to respond fully to the points raised, we would be pleased to receive a revision of your paper within 30 days.

Thank you for submitting your work to EJN.

Kind regards,

John Foxe & Paul Bolam
co-Editors in Chief, EJN

Reviews:

Reviewer: 1

Comments to the Author
The study is clear and straightforward. Indeed, I have only one minor concern.
The DMI is very similar to the RHI and the latter occurs because vision is given a higher weight with respect to touch/proprioception. This phenomenon is consistent with several neuroscientifoc evidence (i.e., vision is a special sense). According to these considerations, I wonder whether the authors could speculate (at least) on the DMI. In this case, the own (voluntary) touch wins over other's touch/proprioception. Any hints around such solution of a multisensory conflict and is action involved?.

Reviewer: 2

Comments to the Author
This series of experiments is about a "basic and primitive motor action" (first line of authors' "Introduction"), i.e. the movement of the hand towards the mouth. The rationale behind the experiments is that few studies have investigated the representation / perceived position of immobile body parts (but have rather looked at the hands, who are incomparably less fixed in space). The present study wanted to fill this gap.
With a novel tactile-proprioceptive illusion, the "dental model illusion" (DMI; the principle being equivalent to the famous RHI, rubber hand illusion, specifically its blind, self-touch version), the authors have explored the perceived location of the mouth, how flexible this percept is and what the relative importance of temporal synchrony vs. spatial congruency is (A total of 6 experiments are described).
Subjects, task(s) and procedures are well-described, the rational of each single experiment is made clear, so technical quality of the work is fine. Laudably, there is a test for the "spread of the effect to other body parts, i.e. from teeth to tip of the nose (exp. 1 and exp. 3). I wonder whether Exp. 6 was truly necessary, the Velcro-detection may be important for the sake of the argument, but otherwise being a rather trivial performance (see also the Figure).
I think the whole series of experiments is a nice extension of what we know about tactile-proprioceptive illusions regarding hand location relative to the body. As the authors state in the Introduction, teeth localization is in many respects different from hand localization (e.g. moveability) and there is some speculation in the literature about an especially early formation (if not "innateness", see below) of the mouth representation. However, as clear the results are, as trivial they seem in retrospect: who would have predicted that a DMI would NOT show up if one properly tested for it? (Same for expectations about micro- and macrogeometric aspects of teeth). While methods, statistics, data visualization etc. are certainly given, the report may be within the scope of the broader readership of the EJN, but it may perhaps disappoint

those readers specialized in cognitive science or behavioral neurology when it comes to the novelty of the findings.
What the authors may consider:

(1) Clearly false is the statement "…phantom pain in persons with congenital absence of limb has been taken as evidence for an innate representation of the limbs…" (p. 5). Please replace "pain" by "sensation".

(2) p. 19: Comparison with the Pinocchio illusion does not make sense. In this illusion a physiological process is used to "dislocate" the finger, whose touch is still felt in the nose (and, consequently, the nose is felt longer). Here, in the DMI, it is the spatial-temporal synchrony of touch which "displaces" a tooth, and it is not surprising that the tip of the nose would follow this displacement (and also ot expected on the basis of the RHI). If the authors want to maintain this comparison, they must justify in what respect the two illusions are similar.

(3) p. 22: I don't see why this report's results would challenge the view that the head is usually considered the "seat of the self". Even if plastic, the spatial position of the mouth may certainly be close to where the self is preferably located (behind the front). As the authors' finding that the nose does NOT shift with the induced shift of a tooth shows, the front (or just the upper part of head/face) can be considered an independent "seat of the self".

(4) The issue of "Innate" and "acquired in utero" (p. 2, but also pp. 5-6 and p. 22)
The authors state that in the literature it would often be assumed that "experience dependent plasticity of spatial representation, which is so characteristic of the brain's hand representation, should be entirely absent for the mouth." (p. 5-6) Although I understand that, in the previous literature, not much sensitivity is present when it comes to the use of the term "innate", the authors' point here seems a bit picky. In fact, all what is present at birth may be designated as "innate" by an author, for whom the whole prenatal life simply does not seem to exist. So, the argument is valid and should be mentioned, but to tacitly assume that previous authors using the term "innate" would have implied "genetically determined" cannot necessarily be justified. It may be due to a neglect of the richness of fetal experience.

(5) I don't understand the brief allusion to autotopagnosia (p. 22) in connection with the representation of specifically the spatial representation of the mouth. Do the authors want to imply that mouth autotopagnosia would be rarer (than, e.g., knee autotopagnosia) because of the evolutionary significance of mouth-related feeding? (the feeding apraxia note is very interesting!)

(6) p. 17: "Neither the main effect of experiment (…), nor the interaction of these two factors were not statistically significant ($F(3,28) = \ldots$)" "not" needs to be removed.

Reviewer: 3

Comments to the Author
Bono and Haggard have submitted a very interesting and original manuscript on a multisensory illusion involving the perceived position of the teeth and the mouth. The authors introduce a novel paradigm – the Dental Model Illusion (DMI) – to investigate the dynamic flexibility of the spatial representation of the teeth and mouth. The paper is well written and includes data from six experiments in which different questions are addressed, including the identification of various temporal, spatial and other congruent factors that

determine the DMI. The design is appropriate, and some of the results are significant. The conclusions are often but perhaps not always supported by the data (see below). My overall assessment is that this is an interesting and original study that will make a valuable contribution to the field. The findings will also be relevant for experimental cognitive dentistry research into dental prostheses.

Major points

1. The authors conclude that their results inform us about the localization of the mouth and the experience-dependent updating of the mouth position. However, this conclusion is probably not supported by the data because the proprioceptive drift tests the perceived location of the maxillary central incisor, not the mouth. Furthermore, the questionnaire results examine the illusory sensation of touching the teeth and provide no information regarding the rest of the mouth. While an argument can be made for why these teeth can be seen as part of the mouth, this issue is unfortunately not discussed in the text. An alternative possibility is that the current results inform us about the representation of the teeth. The authors need to revise their text so their main conclusions are supported by the results. Alternatively, they could discuss this issue and explain to the reader how their "teeth results" inform us about the representation of the mouth. A third, and perhaps the preferred option, would be to conduct a new experiment in which the participants are asked to indicate both the location of the maxillary central incisors ("proprioceptive teeth drift") and of the mouth ("proprioceptive mouth drift"), perhaps by indicating the center of the mouth.

2. In a point related to the first point, the present proprioceptive drift results for the maxillary central incisors could indicate illusory elongation of these teeth, illusory dislocation of the teeth (but not the mouth), or a shift in the location of the mouth and teeth. In the current version of the manuscript, the authors only discuss the last possibility, but I believe they need to explicitly consider the other possibilities as well.

3. I was very surprised to see the lack of a significant effect of spatial congruence in Experiment 3. I think the authors should discuss this further. What do they think could be the reason for this lack of an effect? Perhaps they could speculate why the incongruency manipulation was ineffective. In previous rubber hand illusion experiments, significant effects of spatial congruency-incongruency manipulation have often been found (e.g., Costantini M, Haggard P 2007; Gentile et al. 2013; Guterstam et al. 2011, 2013).

4. It was a positive aspect of the study that there was a large number of experiments, but I was surprised that each experiment only had 8 participants. That is a much smaller sample size than in most of the previous studies on the rubber hand illusion, which have typically used groups of 10-30 participants. You conducted a power analysis, but with 80% power, you still run a reasonable risk of obtaining a false negative result in one of your experiments. I would have recommended having at least 20 participants in each experiment. For example, how can we trust the negative finding in experiment 3 with only 8 participants? Will such a small sample size be sufficient to detect potentially smaller effects related to microgeometric congruencies or spatial congruencies? As a related point, it would be advisable to provide more information about the pilot study that is mentioned on page 9; for example, which conditions did you use, and how was the drift test conducted?

Minor points

5. Introduction, page 4. The authors state that visual-proprioceptive adaptation during the rubber hand illusion leads to a mislocalization of "the tactile percept". However, the proprioceptive drift test probes the position sense of hand location and not the location of the brushstrokes (referral of touch). Please revise the

text for clarity accordingly.

6. Introduction, page 6, second paragraph, the sentence “The idea of innate representation underlying bodily experience receives support from two main areas of the literature”. I agree that the idea of innate body representation is grounded in literature, but there is also evidence - maybe worth mentioning - of the opposite, that body representation is shaped by sensory experience, like in the blind, examined using somatic rubber hand illusion (e.g., Petkova, Zetterberg & Ehrsson, 2011 PLOS ONE; Nava, Steiger & Röder, 2015 Sci Rep), or the plastic effects caused by blindfolding (Radziun and Ehrsson 2018; Sci Rep). Moreover, in this paragraph, when you present evidence in favor of the idea that the mouth representation might be particularly “innate” you refer to phantom limbs. Why do you note cite work that report phantom experiences from teeth, tongue and jaw? Surely such citations would be more relevant to the point that you are trying to make?

7. Introduction page 6. I think the list of the three research questions was not clear. For example, the second and third points are not easy to understand. I think it would be better to replace these three bullet points with a short paragraph in which you provide more explanation of each point, present the specific hypotheses, and outline the experimental strategy used to address each question.

8. Methods, page 7. In the proprioceptive drift test, the participants are instructed to indicate the location of their “maxillary central incisors”. Why are they asked to locate two teeth instead of one specific tooth or the central location between the two maxillary central incisors? Please clarify this choice.

9. Methods, page 12, lines 3-4. Please remove the sentence “Experiment 6 was a manipulation check to experiment 4”. This sentence does not belong in its current location, and it is repeated further below in the text in the paragraph in which you describe Experiment 6.

10. Methods, page 13. Please include an explanation of the motivation for the choice of parametric statistics and include information about how you tested the normality assumptions in your data.

11. Methods, page 14. You state that body ownership can be quantified with proprioceptive drift and cite two papers from the Haggard group. However, this indirect measure is not entirely unproblematic. Under certain conditions the proprioceptive drift does not correlate with subjective ratings of ownership. I recommend adding a sentence or two regarding the strengths and weaknesses of this measure. See for example Abdulkarim and Ehrsson 2016 Atten Percept Psychophys and Rohde et al. 2011 PLOS ONE for recent critical discussions of the proprioceptive drift measure. This is particularly relevant for the current paper where you sometimes get different results from the questionnaires and the proprioceptive drift.

12. Results, page 14, last paragraph. Please add a sentence or two describing the results of Experiments 3, 4 and 5. Even if the results are nonsignificant, it would be informative to the reader to have this information.

13. Results, page 15. A statistical comment. You use t-tests and ANOVAs to analyze questionnaire data even though the Likert scale is ordinal. Ideally, you should use non-parametric statistical tests instead. However, I assume you prefer to use the ANOVA for the 2x2 design and I am sympatric to this approach. But I think you need to add a sentence or two where you provide a justification for your choice of parametric statistics. Just out of curiosity: are your key questionnaire results significant also if you use a non-parametric test?

14. Results, top of page 16. Why are you discussing the significant ANOVA result for the control in statements 2-5? Are these changes in suggestibility and task compliance for the conditions interesting?

15. General discussion, page 18, line 14. In the previous sentence, you explained that there was no significant effect of spatial congruency. Therefore, it seems inconsistent that then conclude that the illusion depends on "the spatiotemporal pattern of tactile stimulation".

16. Discussion on the last paragraph of page 18, the first paragraph of page 19, and page 22. This comment is related to the first major point above. Could it be that the illusion was associated with illusory teeth elongation, illusory teeth displacement, illusory changes in the jaw posture, or forward rotation of the head, rather than changes in the spatial location of the mouth? Can we truly conclude that the spatial representation of the mouth is dynamic given that you only have questionnaire and drift data regarding the maxillary central incisors?

17. Discussion, a comment that is also somewhat related to major points 1-2 above. The authors might want to add a sentence (introduction, methods or discussion) motivating why they call the mouth/teeth localization task for "proprioceptive" drift. Under normal conditions the mouth is immobile with respect to the head. I understand that you might prefer to keep the term "proprioceptive drift" as this is a well-established term in the rubber hand illusion literature, but maybe it would be more appropriate to call it "mouth spatial localization drift" or "mouth localization drift"?

18. Page 19, second paragraph, line 9. In Armel and Ramachandran (2003), the table illusion was as strong as the asynchronous hand illusion and substantially weaker than the synchronous hand illusion. Based on the data presented, I am not convinced that a vivid table illusion was elicited. Please reword this sentence to indicate some caution regarding Armel's and Ramachandran's conclusions. Also, please cite Tsakiris et al. Exp Brain Res 2010 regarding the lack of robust illusions of noncorporal objects.

19. Regarding the issue of microgeometric and macrogeometric experiences and the congruence of surface texture (Discussion, page 20-21), I think you should probably cite the following two studies on this topic: (i) Ward J, Mensah A, Junemann K. The rubber hand illusion depends on the tactile congruency of the observed and felt touch. J Exp Psychol Hum Percept Perform. 2015;41(5):1203-1208; (ii) Schutz-Bosbach S, Tausche P, Weiss C. Roughness perception during the rubber hand illusion. Brain and cognition. 2009;70(1):136-144

20. In the introduction or discussion, there needs to be a clarification that there is no sensory innervation of the surface of the teeth. The sensations we experience at the tips and on the surfaces of the teeth are projected from sensations arising from in the jaw, gum and roots of the teeth. Thus, our default sensory experience of our teeth is fundamentally illusory in nature. Could this fact influence the present results? For example, could the spatial representations of the teeth be more flexible than those of other body parts? Could it be more correct to consider the teeth as "tool extensions" of the gum and jaw rather than "body parts"? If so, could this affect any of the presented results, e.g., the negative finding in Experiment 3?

21. Given that EJN is a neuroscience journal, I would recommend including a brief paragraph (or at least a couple of sentences) in the discussion about the possible neural mechanisms underlying the DMI.

22. The authors should consider citing this article: Perception. 2014;43(8):818-24. The Butcher's Tongue Illusion. Michel C, Velasco C, Salgado-Montejo A, Spence C.

EJN European Journal of Neuroscience FENS Federation of European Neuroscience Societies

Authors' Response 04-Mar-2019

Authors's responses to reviewers

(Changes to the main manuscript are underlined)

Reviewer: 1

Comments to the Author

The study is clear and straightforward. Indeed, I have only one minor concern. The DMI is very similar to the RHI and the latter occurs because vision is given a higher weight with respect to touch/proprioception. This phenomenon is consistent with several neuroscientific evidence (i.e., vision is a special sense). According to these considerations, I wonder whether the authors could speculate (at least) on the DMI. In this case, the own (voluntary) touch wins over other's touch/proprioception. Any hints around such solution of a multisensory conflict and is action involved?.

Thank you for raising this very interesting point. In order to address your comment, we have added the following paragraph to p. 20/21 of the manuscript.

On this view, the DMI might simply reflect Bayesian integration between the signals generated on the two sensory surfaces (the teeth, stimulated by the experimenter) and the finger (stimulated by its movement across the dental model). In RHI, the proprioceptive drift is interpreted in terms of the high weighting given to vision of hand position. In DMI, by analogy, the drift towards the model would indicate a high weighting for the information generated by the haptic exploration of the model by the participant's guided finger movement. We speculate that experience of feeling the dental model as part of one's own body arises as a result of adaptation of teeth position information by finger position information. Where might this adaptation occur? The secondary somatosensory cortices seem to maintain an on-line representation of the body (Press et al., 2008; Tsakiris et al., 2007a ; Tsakiris et al., 2007b) , and are thus a plausible candidate. Posterior parietal cortices might also contribute. This area plays a critical role in representing spatial relations between body-parts (Buxbaum & Coslett, 2001).

Reviewer: 2

Comments to the Author

This series of experiments is about a “basic and primitive motor action” (first line of authors’ “Introduction”), i.e. the movement of the hand towards the mouth. The rationale behind the experiments is that few studies have investigated the representation / perceived position of immobile body parts (but have rather looked at the hands, who are incomparably less fixed in space). The present study wanted to fill this gap.

With a novel tactile-proprioceptive illusion, the “dental model illusion” (DMI; the principle being equivalent to the famous RHI, rubber hand illusion, specifically its blind, self-touch version), the authors have explored the perceived location of the mouth, how flexible this percept is and what the relative importance of temporal synchrony vs. spatial congruency is (A total of 6 experiments are described).

Subjects, task(s) and procedures are well-described, the rational of each single experiment is made clear, so technical quality of the work is fine. Laudably, there is a test for the “spread of the effect to other body parts, i.e. from teeth to tip of the nose (exp. 1 and exp. 3). I wonder whether Exp. 6 was truly necessary, the Velcro-detection may be important for the sake of the argument, but otherwise being a rather trivial performance (see also the Figure).
I think the whole series of experiments is a nice extension of what we know about tactile-proprioceptive illusions regarding hand location relative to the body. As the authors state in the Introduction, teeth localization is in many respects different from hand localization (e.g. moveability) and there is some speculation in the literature about an especially early formation (if not “innateness”, see below) of the mouth representation. However, as clear the results are, as trivial they seem in retrospect: who would have predicted that a DMI would NOT show up if one properly tested for it? (Same for expectations about micro- and macrogeometric aspects of teeth). While methods, statistics, data visualization etc. are certainly given, the report may be within the scope of the broader readership of the EJN, but it may perhaps disappoint those readers specialized in cognitive science or behavioral neurology when it comes to the novelty of the findings.

What the authors may consider:

(1) Clearly false is the statement “…phantom pain in persons with congenital absence of limb has been taken as evidence for an innate representation of the limbs…” (p. 3). Please replace “pain” by “sensation”.

Thank you for pointing out this helpful comment. We have made the following change on p. 3 of the manuscript: the word “pain” has been replaced by “sensation”.

(2) p. 19: Comparison with the Pinocchio illusion does not make sense. In this illusion a physiological process is used to "dislocate" the finger, whose touch is still felt in the nose (and, consequently, the nose is felt longer). Here, in the DMI, it is the spatial-temporal synchrony of touch which "displaces" a tooth, and it is not surprising that the tip of the nose would follow this displacement (and also ot expected on the basis of the RHI). If the authors want to maintain this comparison, they must justify in what respect the two illusions are similar.

Thanks for raising this useful point. We do agree that DMI is conceptually different if compared with the Pinocchio illusion. Perhaps the comparison would be clearer and more accurate if we mentioned the phantom nose illusion instead. This illusion depends on spatial-temporal synchrony of the two touches (one on the nose of the participant and one on another subject's nose). As the participant's movements and their finger contact with the other subject's nose are synchronous with the touch they receive on their nose, the participant experiences the illusion of nose elongation. We did not find evidence for this kind of coherence-preserving transfer across linked body parts in the DMI. Rather, a modular process was followed as the perceived position of teeth (and not their size) is influenced by DMI. Consequently, we applied the following changes to page 17 of the manuscript:

The sentence "For example, in the "Pinocchio illusion", a vibration-induced illusion of elbow extension produced a subjective elongation of the nose if the participant's fingers gripped the tip of their nose." has been replaced by "For example, the "Phantom Nose Illusion" (Hirstein and Ramachandran, 1998) depends on the correlation between two tactile events. One touch is delivered by the experimenter's finger on the participant's nose. The other touch consists in the participant's finger touching the nose of another subject placed just in front of them. As the two touches are synchronous, the participant experiences a subjective sensation of nose elongation. We did not find evidence for this kind of coherence-preserving transfer between linked body parts in the DMI. Rather, a modular organisation (Haggard & Wolpert, 2005) was found, in which local distortions in body representation might be inconsistent with representation of nearby body parts."

(3) p. 22: I don't see why this report's results would challenge the view that the head is usually considered the "seat of the self". Even if plastic, the spatial position of the mouth may certainly be close to where the self is preferably located (behind the front). As the authors' finding that the nose does NOT shift with the induced shift of a tooth shows, the front (or just the upper part of head/face) can be considered an independent "seat of the self".

The reviewer is right in raising this point. The egocentre is a concept which is often discussed but is actually difficult to precisely locate within the body. We modified the manuscript on page 20 to highlight that our findings challenge the view that the spatial location of some non-movable body parts is fixed, by showing that it is experience dependent. The changes applied to the manuscript are the following:

The sentence "Humans tend to consider their head as the seat of their self, the stable core of their body" has been replaced by "The relative positions of some body parts are fixed (e.g., nose and eyes), while the relative positions of other body parts vary frequently (e.g., hands and eyes). The head is often considered as the stable centroid of bodily experience (Alsmith & Longo, 2014). The experiments reported in this article challenge this model by showing that the perceived position of teeth is not stable, but is subject to rapid plastic change through experience."

(4) The issue of "Innate" and "acquired in utero" (p. 2, but also pp. 5-6 and p. 22) The authors state that in the literature it would often be assumed that "experience dependent plasticity of spatial representation, which is so characteristic of the brain's hand representation, should be entirely absent for the mouth." (p. 5-6) Although I understand that, in the previous literature, not much sensitivity is present when it comes to the use of the term "innate", the authors' point here seems a bit picky. In fact, all what is present at birth may be designated as "innate" by an author, for whom the whole prenatal life simply does not seem to exist. So, the argument is valid and should be mentioned, but to tacitly assume that previous authors using the term "innate" would have implied "genetically determined" cannot necessarily be justified. It may be due to a neglect of the richness of fetal experience.

We understand the concern of the reviewer here. For more completeness we have rephrased the following sentence on p.3. "An important tradition in developmental psychology suggests that the perceived position of the mouth may be specified by an innate body schema, since accurate hand to mouth coordination emerges early in infancy". The updated version of the sentence is "An important tradition in developmental psychology suggests that the perceived position of the mouth may be specified by a fixed body schema acquired before birth, since accurate hand to mouth coordination emerges early in infancy". In this way we made sure to not neglect the richness of fetal experience as the reviewer suggested.

(5) I don't understand the brief allusion to autotopagnosia (p. 22) in connection with the representation of specifically the spatial representation of the mouth. Do the authors want to imply that mouth autotopagnosia would be rarer (than, e.g., knee autotopagnosia) because of the evolutionary significance of mouth-related feeding? (the feeding apraxia note is very interesting!)

Thanks for the positive feedback. Our point here is a speculation on the causes behind autotopoagnosia rather than on its incidence on the population. More specifically, we pointed out that when patients face difficulties with independent feeding, that might not only be due to motor weakness. Poor representation of mouth position might play a crucial role in self feeding impairment too.

(6) p. 17: "Neither the main effect of experiment (…), nor the interaction of these two factors were not statistically significant ($F(3,28)$ =…)" "not" needs to be removed.

Thanks for pointing out this typing error. The word "not" has been removed from the aforementioned paragraph on page 15.

Reviewer: 3

Comments to the Author

Bono and Haggard have submitted a very interesting and original manuscript on a multisensory illusion involving the perceived position of the teeth and the mouth. The authors introduce a novel paradigm – the Dental Model Illusion (DMI) – to investigate the dynamic flexibility of the spatial representation of the teeth and mouth. The paper is well written and includes data from six experiments in which different questions are addressed, including the identification of various temporal, spatial and other congruent factors that determine the DMI. The design is appropriate, and some of the results are significant. The conclusions are often but perhaps not always supported by the data (see below). My overall assessment is that this is an interesting and original study that will make a valuable contribution to the field. The findings will also be relevant for experimental cognitive dentistry research into dental prostheses.

Major points

1. The authors conclude that their results inform us about the localization of the mouth and the experience-dependent updating of the mouth position. However, this conclusion is probably not supported by the data because the proprioceptive drift tests the perceived location of the maxillary central incisor, not the mouth. Furthermore, the questionnaire results examine the illusory sensation of touching the teeth and provide no information regarding the rest of the mouth. While an argument can be made for why these teeth can be seen as part of the mouth, this issue is unfortunately not discussed in the text. An alternative possibility is that the current results inform us about the representation of the teeth. The authors need to revise their text so their main conclusions are supported by the results. Alternatively, they could discuss this issue and explain to the reader how their "teeth results" inform us about the representation of the mouth. A third, and perhaps the preferred option, would be to conduct a new experiment in which the participants are asked to indicate both the location of the maxillary central incisors ("proprioceptive teeth drift") and of the mouth ("proprioceptive mouth drift"), perhaps by indicating the center of the mouth.

We are grateful for this point, which we interpret as largely semantic. We agree that our research focusses on the position of the teeth. Teeth are intrinsically part of the mouth, and a landmark for the

EJN European Journal of Neuroscience FENS Federation of European Neuroscience Societies

mouth. "Mouth" itself is an umbrella or system term. Since the mouth is a cavity, the "centre of the mouth" could be a problematic construct – it might not be a body part but empty space. Since our study falls within the general experimental topic of somatosensory body representation, we wanted to define the position of the "mouth" in terms of the position within the body of some appropriate sensitive tissue. The receptors that make teeth sensory organs sensitive to touch have their physical seat not on dental surfaces, but inside the mouth itself. More specifically they are located on PDL, fibers that connect teeth to the gum. Many classic RHI experiments stimulated the back of the hand and asked participants to judge the position of the index fingertip – therefore, some dissociation between the stimulation and the psychological construct/dependent variable seems reasonable. The following sentence has been added to p.19 to clarify this point:

<u>Interestingly, the receptors that underlie mechanosensitivity of the teeth are located on periodontal ligaments, which attach the root of the tooth to the alveolar bone (Trulsson & Johansson, 1996; Trulsson & Johansson, 2002). Thus, the perceived position of the teeth is directly linked, at receptor level, to the perceived position of the upper jaw itself. However, the perceived position of the nose appeared to be handled separately.</u>

2. In a point related to the first point, the present proprioceptive drift results for the maxillary central incisors could indicate illusory elongation of these teeth, illusory dislocation of the teeth (but not the mouth), or a shift in the location of the mouth and teeth. In the current version of the manuscript, the authors only discuss the last possibility, but I believe they need to explicitly consider the other possibilities as well.

At the end of each trial, each participant completed the DMI questionnaire. In this questionnaire only the first statement was designed to correspond to the illusion. The other four were control statements for suggestibility. More specifically, statement 3 specifically investigated whether DMI could induce a perceived elongation of teeth by asking participants whether they perceived their teeth to be modified in their size after tactile illusion. As experiments 1-5 show, the average rating given to this statement after synchronous conditions is statistically comparable to the average rating given after asynchronous tactile stimulation. That is also the case when the dental model is modified in either its microgeometric (experiment 4) or macrogeometric (experiment 5) properties. Consequently, we could rule out the hypothesis that DMI might be explained by illusory elongation of teeth. We have added the following brief paragraph to p.17 of the discussion to highlight that this appears to be a change in perceived position, not perceived size:

<u>For example, item 3 of the DMI questionnaire specifically investigated whether DMI could induce a perceived elongation of teeth by asking participants whether they perceived their teeth to be modified in their size after tactile illusion. In principle the different spatial locations of the afferent information from the participant's teeth and from their fingertip could be reconciled if the teeth were perceived as</u>

longer than their true length (de Vignemont et. al, 2005). As experiments 1-5 show, the average rating given to this statement did not differ between synchronous and asynchronous conditions. Consequently, the perceived position of the teeth was influenced by DMI, but the size of the teeth was not. This result suggests that local inconsistencies are tolerated when multisensory stimulation drives plastic adaptations of body representation. The perceived position of the upper incisors changed, without any comparable change in the perceived position of the nose – despite the fixed anatomical relation between these two body parts.

3. I was very surprised to see the lack of a significant effect of spatial congruence in Experiment 3. I think the authors should discuss this further. What do they think could be the reason for this lack of an effect? Perhaps they could speculate why the incongruency manipulation was ineffective. In previous rubber hand illusion experiments, significant effects of spatial congruency-incongruency manipulation have often been found (e.g., Costantini M, Haggard P 2007; Gentile et al. 2013; Guterstam et al. 2011, 2013).

First, we would urge caution in interpreting the null result. We found no spatial congruence effect – but this could of course be a matter of statistical power, particularly since our studies are individually quite small. Costantini and Haggard (2007) showed that congruency modulations did not abolish the RHI when the participant's hand was out of sight. This may be because spatial precision of tactile orientation judgement is quite poor, implying that congruence effects would be weak. Hence modulations of temporal aspects of the stimulation (asynchronous stroking, see experiments 1,2, 4, and 5) are necessary to significantly reduce DMI. We have added a brief comment and reference to Costantini to p.16/17 of the discussion as follows:

Costantini and Haggard (2007) showed that spatial incongruency of up to 30° between the orientation of viewed and felt stroking movements did not abolish the RHI. Importantly, in their study the participant's hand was out of sight, so the incongruency may simply have been undetected. Our findings, in the case of teeth stimulation, again suggest that synchronised sensory inputs can cause illusions of bodily awareness even when the spatial features of the two sensory inputs differ.

4. It was a positive aspect of the study that there was a large number of experiments, but I was surprised that each experiment only had 8 participants. That is a much smaller sample size than in most of the previous studies on the rubber hand illusion, which have typically used groups of 10-30 participants. You conducted a power analysis, but with 80% power, you still run a reasonable risk of obtaining a false negative result in one of your experiments. I would have recommended having at least 20 participants in each experiment. For example, how can we trust the negative finding in experiment 3 with only 8 participants? Will such a small sample size be sufficient to detect potentially smaller effects related to microgeometric congruencies or spatial congruencies? As a related point, it would be advisable to provide

more information about the pilot study that is mentioned on page 9; for example, which conditions did you use, and how was the drift test conducted?

We are grateful for this point. Yes, the studies are individually small. However, we did perform a power calculation, and we did perform multiple close replications. Both of these methods are key tools in modern scientific research methods and seem more in keeping with current best practice than the rules of thumb regarding sample size that have traditionally prevailed in particular research areas. Recent methods research (Kenny & Judd, 2019) suggests that multiple small studies may be more valuable than a single large study when effect sizes are heterogenous (which one might expect here given the slightly varying experimental design and research question).
Furthermore, additional details regarding the pilot have been added to page 8/9 as follows:

Pilot testing consisted of three phases: pre-test position estimation, 60s of synchronous tactile stimulation and post-test position estimation. The procedure used during these three phases was identical to Experiments 1-5.

Minor points

5. Introduction, page 4. The authors state that visual-proprioceptive adaptation during the rubber hand illusion leads to a mislocalization of "the tactile percept". However, the proprioceptive drift test probes the position sense of hand location and not the location of the brushstrokes (referral of touch). Please revise the text for clarity accordingly.

Thanks for the comment. Any mention to tactile/visual percept which might result ambiguous for interpretation has been removed from p.4 of Introduction.

6. Introduction, page 6, second paragraph, the sentence "The idea of innate representation underlying bodily experience receives support from two main areas of the literature". I agree that the idea of innate body representation is grounded in literature, but there is also evidence - maybe worth mentioning - of the opposite, that body representation is shaped by sensory experience, like in the blind, examined using somatic rubber hand illusion (e.g., Petkova, Zetterberg & Ehrsson, 2011 PLOS ONE; Nava, Steiger & Röder, 2015 Sci Rep), or the plastic effects caused by blindfolding (Radziun and Ehrsson 2018; Sci Rep). Moreover, in this paragraph, when you present evidence in favour of the idea that the mouth representation might be particularly "innate" you refer to phantom limbs. Why do you note cite work that report phantom experiences from teeth, tongue and jaw? Surely such citations would be more relevant to the point that you are trying to make?

That is a really good point. The manuscript has been updated by adding the following paragraph to page 5 of the manuscript.

However, other studies have highlighted the role of sensory experience in body representation by showing that blind individuals have a less dynamic multisensory representation of their own limbs. (Nava et al., 2014; Petkova et al., 2012).

Regarding the second part of the comment, in the aforementioned paragraph we argued how previous literature supported the idea of innate body representation. Therefore, we mentioned cases of phantom pain in persons with congenital absence of limb. Tooth pain cases have often been reported in literature (e.g. Marbach, 1993). However, all these cases show phantom pain after sensory deafferentation (tooth extraction, pulp extirpation, etc.). To our best knowledge, no studies have yet reported cases of phantom pain syndrome in persons with congenital absence of a given tooth cluster. Hence such clinical cases can be mentioned as evidence of persistent body representation even after deafferentation. However, we advise that they cannot be mentioned as an example of innate body representation as these individuals have been exposed to multiple sensory experiences through teeth before deafferentation.

7. Introduction page 6. I think the list of the three research questions was not clear. For example, the second and third points are not easy to understand. I think it would be better to replace these three bullet points with a short paragraph in which you provide more explanation of each point, present the specific hypotheses, and outline the experimental strategy used to address each question.

Thanks for raising this point. We have removed the list of three bullet points. A paragraph highlighting hypotheses and experimental strategies of each experiment has been added to p. 5 and 6 as follows:

We first investigated whether correlated tactile and proprioceptive experience is sufficient to elicit changes in the perceived location and sense of ownership with respect to the teeth and the mouth. Accordingly, we manipulated the spatial and temporal aspects of tactile stimulation in Experiment 1 and 2, and spatial aspects only in Experiment 3. Second, we investigated whether mouth stimulation causes experience-dependent modulation of the mouth only or also of other body parts. Consequently, in experiments 1 and 3 we asked to judge the perceived position of both stimulated and adjacent non-stimulated body parts. Last, we wanted to understand to what extent top-down knowledge (Tsakiris & Haggard, 2005) about the morphology of one's own teeth influence any experience-dependent plasticity effects. To address this research question, we modified the microgeometric (experiment 4) and macrogeometric aspects (experiment 5) of the dental model used during tactile stimulation.

8. Methods, page 7. In the proprioceptive drift test, the participants are instructed to indicate the location

of their “maxillary central incisors”. Why are they asked to locate two teeth instead of one specific tooth or the central location between the two maxillary central incisors? Please clarify this choice.

Participants received tactile stimulation on both the maxillary central incisors. Consequently, we decided to let them judge the spatial location of both the body parts touched during testing. We reasonably assume that their judgements represented a pooling of information about both these teeth. Note that these two teeth have the same vertical position, and our dependent variable deals with vertical position. Thus, our measures should be independent of the weighting each participant gave to each of the two teeth in responding to our instructions.

9. Methods, page 12, lines 3-4. Please remove the sentence “Experiment 6 was a manipulation check to experiment 4”. This sentence does not belong in its current location, and it is repeated further below in the text in the paragraph in which you describe Experiment 6.

Thanks. The sentence has been removed.

10. Methods, page 13. Please include an explanation of the motivation for the choice of parametric statistics and include information about how you tested the normality assumptions in your data.

To check normality, we proceeded in the following way:

1) In each experiment (1-5), we computed residuals for each testing condition
2) We then tested the normality of distribution of residuals for each condition separately with Shapiro-Wilk test of normality
3) We plotted the normal Q-Q plot

Some experiments (1, 3 and 5) have one condition each which violated the assumption of Normality. In addition, conditions that violated normality had low kurtosis (e.g., k=1.5), perhaps due to outlier scores in these conditions. We ran non-parametric tests and their results are in line with parametric tests. For the sake of completeness, results of non-parametric tests have been reported in the supporting information section. Please see response to comment 13 for further details.

11. Methods, page 14. You state that body ownership can be quantified with proprioceptive drift and cite two papers from the Haggard group. However, this indirect measure is not entirely unproblematic. Under certain conditions the proprioceptive drift does not correlate with subjective ratings of ownership. I recommend adding a sentence or two regarding the strengths and weaknesses of this measure. See for example Abdulkarim and Ehrsson 2016 Atten Percept Psychophys and Rohde et al. 2011 PLOS ONE for

recent critical discussions of the proprioceptive drift measure. This is particularly relevant for the current paper where you sometimes get different results from the questionnaires and the proprioceptive drift.

Thanks for this point. Proprioceptive drift is a pertinent measure of bodily awareness, but it is certainly not unproblematic, and is not an accepted gold standard. We have added a sentence on p.12 to this effect, and referenced the papers mentioned.

This measure has been widely used in the literature on bodily awareness to indicate the strength of multisensory illusions (Longo & Haggard, 2012; Tsakiris & Haggard, 2005). However, some studies have noted that this measure can dissociate from explicit reports, and have therefore questioned it (Abdulkarim & Ehrsson, 2016; Rohde et al., 2011).

12. Results, page 14, last paragraph. Please add a sentence or two describing the results of Experiments 3, 4 and 5. Even if the results are nonsignificant, it would be informative to the reader to have this information.

Thanks. A sentence describing results of experiments 3,4 and 5 has been added on p. 13 as follows:

No significant interactions were observed in the ANOVAs computed in experiments 3,4 and 5.

13. Results, page 15. A statistical comment. You use t-tests and ANOVAs to analyze questionnaire data even though the Likert scale is ordinal. Ideally, you should use non-parametric statistical tests instead. However, I assume you prefer to use the ANOVA for the 2x2 design and I am sympatric to this approach. But I think you need to add a sentence or two where you provide a justification for your choice of parametric statistics. Just out of curiosity: are your key questionnaire results significant also if you use a non-parametric test?

Thanks for your comment. Data in experiments 2 and 4 are normally distributed so we used parametric tests to analyse the data of these experiments. Experiments 1,3 and 5 have one condition each which is not normally distributed. Therefore, we firstly analysed data of these experiments using non parametric tests. We also ran parametric tests as they are more discriminating by allowing a more in depth exploitation of observed results (Mircioiu & Atkinson, 2017). Since parametric and non-parametric analyses gave the same significant and non-significant results for inter-subgroup comparisons, we decided to report in the main text results. Results of non-parametric tests are reported in the supporting information section. A new paragraph has been added to p.13 as follows:

All data were assessed for normality using the Shapiro–Wilk test ($p > 0.05$), and the appropriate non-parametric tests were applied when one or more of the corresponding data sets failed to meet the criteria for normal distribution. More specifically, questionnaire data in experiments 1, 3 and 5 were not normally distributed. In these cases, we ran both parametric and non-parametric tests, and found

similar patterns of significance. Parametric tests are more commonly used for factorial designs such as ours (Mircioiu & Atkinson, 2017), we report the parametric tests in the paper, and non-parametric tests for experiments 1, 3 and 5 in supporting information.

14. Results, top of page 16. Why are you discussing the significant ANOVA result for the control in statements 2-5? Are these changes in suggestibility and task compliance for the conditions interesting?

These results were discussed just for the sake of completeness, but they can be easily removed if it needs be.

15. General discussion, page 18, line 14. In the previous sentence, you explained that there was no significant effect of spatial congruency. Therefore, it seems inconsistent that then conclude that the illusion depends on “the spatiotemporal pattern of tactile stimulation”.

Thanks. This is a tricky semantic point of the English language. The illusion does depend on the spatiotemporal pattern of tactile stimulation. As shown by this study, the combination of temporal and spatial modulations (asynchronous stroking) is necessary to statistically reduce DMI (experiments 1,2,4 and 5). DMI survives when only spatial modulations (spatially incongruent stroking that is temporally synchronised) are applied (experiment 3). We believe that the stimulation schematics in the paper (fig.3) make it clear what is meant.

16. Discussion on the last paragraph of page 18, the first paragraph of page 19, and page 22. This comment is related to the first major point above. Could it be that the illusion was associated with illusory teeth elongation, illusory teeth displacement, illusory changes in the jaw posture, or forward rotation of the head, rather than changes in the spatial location of the mouth? Can we truly conclude that the spatial representation of the mouth is dynamic given that you only have questionnaire and drift data regarding the maxillary central incisors?

Please see response to comments 1 and 2 for further details.

17. Discussion, a comment that is also somewhat related to major points 1-2 above. The authors might want to add a sentence (introduction, methods or discussion) motivating why they call the mouth/teeth localization task for “proprioceptive” drift. Under normal conditions the mouth is immobile with respect to the head. I understand that you might prefer to keep the term “proprioceptive drift” as this is a well-established term in the rubber hand illusion literature, but maybe it would be more appropriate to call it “mouth spatial localization drift” or “mouth localization drift”?

This is an interesting point, and again a semantic one. In RHI, Proprioceptive drift might refer to an adaptation of perception of input from proprioceptive afferents – the hand can move relative to the rest of the body, and the proprioceptive afferents may signal that fact. The teeth do not move relative to the head in the same way. However, our use of "proprioceptive drift" is (a) consistent with the RHI literature, to which it is clearly methodologically related (b) etymologically correct, in the sense that it is a drift in the perception of part of one's own body. In DMI, it does not relate to any specific afferent class or body motion, as it does in RHI. However, the term "proprioceptive drift" per se makes no commitment to either afferents or body motions. We take the reviewer's point that the situation is different from RHI, but the term itself is not semantically incorrect in this context, and we see a value in keeping the terminology aligned with the RHI literature.

18. Page 19, second paragraph, line 9. In Armel and Ramachandran (2003), the table illusion was as strong as the asynchronous hand illusion and substantially weaker than the synchronous hand illusion. Based on the data presented, I am not convinced that a vivid table illusion was elicited. Please reword this sentence to indicate some caution regarding Armel's and Ramachandran's conclusions. Also, please cite Tsakiris et al. Exp Brain Res 2010 regarding the lack of robust illusions of noncorporal objects.

Thanks for raising this comment. We believe that the reviewer refers to experiment 2 from Armel and Ramachandran (2003). We do agree that these results must be interpreted with caution. In particular, this experiment shows that the table illusion was as strong as the RHI in case the rubber hand is shown to the participant. Both of them are substantially weaker than the synchronous hand illusion when the real hand is hidden (classic RHI effect). However, Experiment 2 of that study does not compare synchronous VS asynchronous. Therefore, what their results support is that synchronous stroking of table and participant's hand might induce a weak RHI effect. We have modified the second paragraph of page 18 accordingly by highlighting the weak nature of the table effect.

For example, they found that people could develop a weak RHI effect when a tabletop was stroked in synchrony with their own hand. However, caution is required in interpreting these data as other studies did not find any reliable illusion for non-body objects (Tsakiris et al., 2010).

Regarding the second point of the comment, thanks for the suggested citation. We have added it to the reference list.

19. Regarding the issue of microgeometric and macrogeometric experiences and the congruence of surface texture (Discussion, page 20-21), I think you should probably cite the following two studies on this topic: (i) Ward J, Mensah A, Junemann K. The rubber hand illusion depends on the tactile congruency of the observed and felt touch. J Exp Psychol Hum Percept Perform. 2015;41(5):1203-1208; (ii) Schutz-Bosbach S, Tausche P, Weiss C. Roughness perception during the rubber hand illusion. Brain and cognition. 2009;70(1):136-144

We really appreciate this useful comment. We have added the following sentence to p. 19 of the manuscript:

These findings are in line with the RHI literature, which shows that RHI does not depend on conceptual interpretation of tactile sensations (Schütz-Bosbach et al., 2009).

20. In the introduction or discussion, there needs to be a clarification that there is no sensory innervation of the surface of the teeth. The sensations we experience at the tips and on the surfaces of the teeth are projected from sensations arising from in the jaw, gum and roots of the teeth. Thus, our default sensory experience of our teeth is fundamentally illusory in nature. Could this fact influence the present results? For example, could the spatial representations of the teeth be more flexible than those of other body parts? Could it be more correct to consider the teeth as “tool extensions” of the gum and jaw rather than “body parts”? If so, could this affect any of the presented results, e.g., the negative finding in Experiment 3?

This is an interesting point, but again it is almost a semantic one. We agree that teeth sensation is not directly generated on the tactile surface. But what sensations are truly direct? Proprioceptive sensations are generated at joints and tendons and muscles, but may be perceived as hand positions. Tactile sensations may be generated deep within the dermis, rather than at the skin surface. Teeth do not have receptors on their surface, but they are connected with tissues that contain receptors. The most similar analogous on the human body is represented by nails. Previous studies have investigated such cases of indirect perception (Seah, Wu, Sebastin, & Lahiri, 2013).
Results showed that tactile discrimination on indirect sensory organs (e.g. nails) is directly comparable to the performance obtained on direct sensory organs (e.g. fingertip). Tools, in contrast, are not normally physically connected to the human body. To us, the Periodontal Ligaments Mechanoreceptors seem like a more direct case than a tool – but it may well be a matter of degree. We have added a sentence in the discussion (p.18) about directness and referred sensation, but we see this as almost a semantic point. Please see our reply to comment 2 for further details.

Interestingly, the receptors that underlie mechanosensitivity of the teeth are located on periodontal ligaments, which attach the root of the tooth to the alveolar bone (Trulsson & Johansson, 1996; Trulsson & Johansson, 2002).

21. Given that EJN is a neuroscience journal, I would recommend including a brief paragraph (or at least a couple of sentences) in the discussion about the possible neural mechanisms underlying the DMI.

We really appreciated that you raised this comment. We addressed this point in the reply given to comment 1 from reviewer 1. Any consequent change to the manuscript is highlighted in our reply to that comment.

22. The authors should consider citing this article: Perception. 2014;43(8):818-24. The Butcher's Tongue Illusion. Michel C, Velasco C, Salgado-Montejo A, Spence C.

This paper was already cited in the version of the manuscript that the reviewer saw.

References

Abdulkarim, Z., & Ehrsson, H. H. (2016). No causal link between changes in hand position sense and feeling of limb ownership in the rubber hand illusion. *Attention, Perception & Psychophysics*, *78*, 707–720. https://doi.org/10.3758/s13414-015-1016-0

Buxbaum, L. J., & Coslett, H. B. (2001). Specialised structural descriptions for human body parts: Evidence from autotopagnosia. *Cognitive Neuropsychology*, *18*(4), 289–306. https://doi.org/10.1080/02643290126172

Costantini, M., & Haggard, P. (2007). The rubber hand illusion: sensitivity and reference frame for body ownership. *Consciousness and Cognition*, *16*(2), 229–240. https://doi.org/10.1016/j.concog.2007.01.001

Kenny, D. A., & Judd, C. M. (2019). The unappreciated heterogeneity of effect sizes: Implications for power, precision, planning of research, and replication. *Psychological Methods*, No Pagination Specified-No Pagination Specified. https://doi.org/10.1037/met0000209

Marbach, J. J. (1993). Is phantom tooth pain a deafferentation (neuropathic) syndrome? Part I: Evidence derived from pathophysiology and treatment. *Oral Surgery, Oral Medicine, and Oral Pathology*, *75*(1), 95–105.

Mircioiu, C., & Atkinson, J. (2017). A Comparison of Parametric and Non-Parametric Methods Applied to a Likert Scale. *Pharmacy: Journal of Pharmacy, Education and Practice*, *5*(2). https://doi.org/10.3390/pharmacy5020026

Nava, E., Steiger, T., & Röder, B. (2014). Both developmental and adult vision shape body representations. *Scientific Reports*, *4*, 6622. https://doi.org/10.1038/srep06622

Petkova, V. I., Zetterberg, H., & Ehrsson, H. H. (2012). Rubber Hands Feel Touch, but Not in Blind Individuals. *PLoS ONE*, *7*(4). https://doi.org/10.1371/journal.pone.0035912

Press, C., Heyes, C., Haggard, P., & Eimer, M. (2008). Visuotactile learning and body representation: an ERP study with rubber hands and rubber objects. *Journal of Cognitive Neuroscience*, *20*(2), 312–323. https://doi.org/10.1162/jocn.2008.20022

Rohde, M., Luca, M. D., & Ernst, M. O. (2011). The Rubber Hand Illusion: Feeling of Ownership and Proprioceptive Drift Do Not Go Hand in Hand. *PLOS ONE*, *6*(6), e21659. https://doi.org/10.1371/journal.pone.0021659

EJN European Journal of Neuroscience FENS Federation of European Neuroscience Societies

Schütz-Bosbach, S., Tausche, P., & Weiss, C. (2009). Roughness perception during the rubber hand illusion. *Brain and Cognition*, *70*(1), 136–144. https://doi.org/10.1016/j.bandc.2009.01.006
Seah, B. Z. Q., Wu, C. C. H., Sebastin, S. J., & Lahiri, A. (2013). Tactile sensibility on the fingernail. *The Journal of Hand Surgery*, *38*(11), 2159–2163. https://doi.org/10.1016/j.jhsa.2013.08.112
Tsakiris, M. (2010). My body in the brain: a neurocognitive model of body-ownership. *Neuropsychologia*, *48*(3), 703–712. https://doi.org/10.1016/j.neuropsychologia.2009.09.034
Tsakiris, M., Hesse, M. D., Boy, C., Haggard, P., & Fink, G. R. (2007). Neural signatures of body ownership: a sensory network for bodily self-consciousness. *Cerebral Cortex (New York, N.Y.: 1991)*, *17*(10), 2235–2244. https://doi.org/10.1093/cercor/bhl131
Tsakiris, M., Schütz-Bosbach, S., & Gallagher, S. (2007). On agency and body-ownership: phenomenological and neurocognitive reflections. *Consciousness and Cognition*, *16*(3), 645–660. https://doi.org/10.1016/j.concog.2007.05.012

2nd Editorial Decision 17-Apr-2019

Dear Mr. Bono,

Your revised manuscript was re-evaluated by external reviewers as well as by the Section Editor, Dr. Sophie Molholm and ourselves. We are pleased to inform you that we expect that it will be acceptable for publication in EJN following further revision and possible re-review. As you will read, two of our expert reviewers are now fully satisfied with your revisions, but the third reviewer, while also acknowledging that you have addressed the majority of concerns raised, questions the strength of the conclusions that you draw on the basis of negative results. You will need to consider modifying/softening interpretation of the findings accordingly. Reviewer 3 also indicates some additional findings in the literature that you might want to take into account in interpreting your data, and suggests an additional analysis. Modifying the tone of your interpretations when they are based on null findings should be done in a revision; the other suggested modifications, while certainly worth considering, are largely interpretive and are left to the authors discretion. Nevertheless, please address the reviewers comments in a response letter.

IMPORTANT: Also, we will not proceed until you replace the barcharts in your figure with scatterplots or some other more informative hybrid depictions - see http://onlinelibrary.wiley.com/doi/10.1111/ejn.13400/epdf

Additional queries are indicated in the comments below.

When revising the manuscript, please embolden or underline major changes to the text so they are easily identifiable and DO NOT leave 'track change' formatting marks in your paper. At this stage, please provide text and a figure file for the Graphical Abstract. When carrying out your revisions please refer to the checklist below and visit the EJN author guidelines at www.ejneuroscience.org

When finalised, please upload your complete revised manuscript onto the website as a Word (.doc or .docx), or .rtf file. Please also ensure that a complete set of tables and figures is included as separate files, even if these have not changed from the originals. At this stage it is necessary to provide high resolution figures. Please see important instructions below.

Please go into https://mc.manuscriptcentral.com/ejn - Author Centre - manuscripts with decisions where you will find a 'create a revision' link under 'actions'. We ask that you please indicate the way in which you have responded to the points raised by the Editors and Reviewers in a letter. Please upload this response letter as a separate Word (.doc) file using the file designation "Authors' Response to Reviewers" when uploading your manuscript files. Please DO NOT submit your revised manuscript as a new one. Also, please note that only the Author who submitted the original version of the manuscript should submit a revised version.

If you are able to respond fully to the points raised, we shall be pleased to receive a revision of your paper within 30 days.

Thank you for submitting your work to EJN.

Kind regards,

John Foxe & Paul Bolam
co-Editors in Chief, EJN

Reviews:

Reviewer: 1

Comments to the Author
(There are no comments.)

Reviewer: 2

Comments to the Author
I am entirely satisfied how you dealt with the points I raised - thank you.

Reviewer: 3

Comments to the Author
Bono and Haggard have done an excellent job of revising the manuscript, and they have answered most of my points in a very satisfactory way. I also like how they dealt with the important points highlighted by reviewers 1 and 2. But some of my original critical comments deserve further discussion and possible further revisions (see below). In particular, this relates to how the authors interpret and present some of their negative results.

(i) Regarding original point 3. I am happy with the way you expanded the discussion about the spatial congruence issue (in the discussion). You could consider to cite the study of Aimola Davies et al (2013, Consciousness and Cognition). These authors found that their version of the somatic rubber hand illusion

was surprisingly tolerant to spatial incongruencies, which is in line with the current findings. However, I think the authors should interpret their negative result with more caution. The p-value was p=0.207 for the questionnaire data and p=0.244 for the proprioceptive drift data. And the N is small. Therefore, a false negative result cannot be excluded. I assume that a Bayesian analysis would not provide strong evidence in favor of the null hypothesis.

(ii) Regarding original point 4. I agree that the basic results that support the existence of the DMI are robust. The metanalyses presented in Figure 7 also nicely corroborates your main conclusion.

However, some of your secondary results could be false negatives; and here the small sample size in the individual experiments becomes a weakness. In particular, I am thinking about the negative findings in Exp 4 and 5 and the conclusion that you presented in the abstract: "DMI was not strongly affected by manipulating the macrogeometric or microgeometric tactile properties of the dental model, suggesting cognitive images of one's own oral morphology play a minimal role". However, the stroking x microgeometry interaction gave a p-value of 0.093, and in Figure 5D it looks like you have a positive result: the drift looks much greater in the congruent synchronous condition compared to the incongruent synchronous condition. Moreover, the questionnaire results for illusion statement 1 (Table 5) specifies a main effect of macrogeometry at p=0.03, and a stroking x macrogeometry interaction of p=0.107. These are not the kinds of results that support a genuine negative finding in my opinion. If you want to present the negative macrogeometric result as a major finding of the study and highlight it in the abstract you should report a Bayes factor in favor of the null hypothesis using a Bayesian statistical analysis.

In addition to this concern, I think your macrogeometric manipulation is quite different from Tsakiris' block of wood condition. You have only removed one tooth, and that would be similar to removing one finger from the rubber hand I would imagine. So, the "top-down representation" effect in your paradigm is probably weaker compared to the classic block of wood condition. This would make the effect you are looking for small, which in turn implies that that experiment is probably underpowered. In sum, I think you should tone down your conclusions regarding a lack of an effect of macrogeometric incongruence, and remove this result from the abstract.

I think the same goes for your strong conclusions regarding microgeometric incongruence in the discussion on page 61: "We conclude that surface texture is not a major modulator of tooth ownership". You should probably be more cautious here given the small N, the lack of Bayesian analysis favoring the null hypothesis, and the fact that not all results point in this direction. Look at the questionnaire result for example. In Table 2, the effect of microgeometry on illusion strength is p=0.080.

(iii) Regarding original point 11. Abdulkarim and Ehrsson (2016) observed a correlation between the sense of hand ownership and proprioceptive drift (shift) towards the rubber hand, but nevertheless, these authors urged caution when using the drift measure because they did not observe changes in hand ownership when they experimentally manipulated proprioception. Therefore, I suggest you reformulate your new sentence slightly:

"However, some studies have noted that this measure can dissociate from explicit reports, and have therefore recommend caution when using it (Abdulkarim & Ehrsson, 2016; Rohde et al., 2011)."

(iv) Regarding original point 21. Good that you have now included a few sentences about DMI's possible neural substrate in the general discussion. But I am only moderately impressed with your neuroanatomical

speculations I have to admit. The posterior parietal cortex is certainly a good candidate, and you could add a couple of references at the end of the sentence where you mention this region (page 62): e.g., Ehrsson et al 2005a J Neurosci and Ehrsson et al. 2005b PLOS Biol. The intraparietal cortex of the posterior parietal cortex is active during the somatic rubber hand illusion (Ehrsson et al 2005a) and during a version of Lackner's "Pinnochi illusion" induced by placing the vibration-stimulated hands against the waist eliciting "illusory waist shrinkage" (Ehrsson et al 2005b). Activity in the posterior parietal cortex also correlates with the proprioceptive drift towards the rubber hand (Brozzoli et al 2012 J Neurosci; a BOLD-adaption index of a shift in perihand space towards the rubber hand predicted the degree of proprioceptive shift towards the rubber hand). The somatic rubber hand illusion also activated the premotor cortex and cerebellum should I add. The premotor cortex could be relevant to mention because the activity in this region correlated with the strength of the self-touch illusion (Ehrsson 2005a). Moreover, in the brain of macaques, both the premotor cortex and the posterior parietal cortex contain extensive multisensory representations of the hand and mouth (Graziano and Cooke 2006 Neuropsychologia). The reasons for this is probably that hand and mouth actions are coordinated during eating behavioral, and that the hand is retracted towards the face during defensive action. Thus, in my opinion it is reasonable to speculate that these fronto-parietal regions could also be involved in the DMI. Regarding the Tsakiris et al (2007) Cerebral Cortex study, these authors reported a correlation between proprioceptive drift and rCBF in the posterior insula (in a fixed-effect analysis across four different conditions). This activation was not located in the secondary somatosensory cortex if I remember correctly (?); but PET has relatively low spatial resolution and posterior insula and secondary somatosensory cortex are adjacent regions that could be difficult to anatomically distinguish. More problematically, in the Tsakiris study it is not clear if the correlation is driven by the proprioceptive drift towards the rubber hand in the synchronous congruent (illusion) condition, as opposed to by differences in drift between the various control conditions. Buxbaum and Coslett (2001) analyzed structural descriptions of the body that involve semantic representation. This type of representation is probably different from the perceptual processes involved in the somato-proprioceptive illusion you are examining. Personally, I would not expect a common neural substrate between DMI and body structure description in the posterior parietal cortex.

(v) New point 1. In Figure 3 what is plotted on the x-axis? I assume it is time but it would be nice to know the scale.

(vi) New point 2. In Figure 3 it is difficult to read the individual statements. Please list the questionnaire statements in a supplementary table or make this figure panel bigger.

(vii) New point. In Table 1 the title says "Experiments 1, 2, 4 and 5" but in the table Experiment 2 is not listed, but Experiment 3.

(viii). Finally, in the previous review I forgot to ask you if you found a significant correlation between proprioceptive drift and questionnaire statement 1. You could compute the proprioceptive shift by subtracting the drift in the asynchronous condition from the drift in the synchronous condition, and then test if this shift correlates with the difference score in illusion strength (statement 1) when subtracting the asynchronous condition from the synchronous condition. You could run this analysis across all 32 subjects to enhance statistical power. In my opinion, observing a significant correlation between drift and subjective ratings would corroborate your main conclusion.

EJN European Journal of Neuroscience FENS Federation of European Neuroscience Societies

Authors's responses to reviewers

(Changes to the main manuscript are underlined)

As pointed out from the editor, barcharts have been removed from figures 5, 6 and 8. Captions of figures 5, 6 and 8 on pages 33-34 of the main manuscript have been updated as follows:

Figure 5. Box plots showing the proprioceptive drift across different stroking conditions in Experiments 1-5. Each single-subject value corresponds to the proprioceptive drift toward the dental model (in cm). In all the box-plots lower and upper box boundaries represent 25th and 75th percentiles respectively and the line inside box shows the median. *p < .05, **p < .01.

Figure 6. Box plots showing the rating to the statements of the Dental Model Illusion Questionnaire obtained in experiments 1-5. Each single-subject value corresponds to the rating to the given DMI questionnaire's statement. In all the box-plots lower and upper box boundaries represent 25th and 75th percentiles respectively and the line inside box shows the median. *p < .05, **p < .01.

Figure 8. 2x3 pooled ANOVAs computed with stroking as within-subject factor and experiment as a between-subject factor on the proprioceptive drift (A) on the rating given to the first statement of the DMI questionnaire (B) and on the pooled rating of statements 2-5 (C). Each single-subject value corresponds to the proprioceptive drift toward the dental model (in cm) (A) and to the rating to the given DMI questionnaire's statement (B and C). In all the box-plots lower and upper box boundaries represent 25th and 75th percentiles respectively and the line inside box shows the median. *p < .05, **p < .01.

Bono and Haggard have done an excellent job of revising the manuscript, and they have answered most of my points in a very satisfactory way. I also like how they dealt with the important points highlighted by reviewers 1 and 2. But some of my original critical comments deserve further discussion and possible further revisions (see below). In particular, this relates to how the authors interpret and present some of their negative results.

(i) Regarding original point 3. I am happy with the way you expanded the discussion about the spatial congruence issue (in the discussion). You could consider to cite the study of Aimola Davies et al (2013, Consciousness and Cognition). These authors found that their version of the somatic rubber hand illusion was surprisingly tolerant to spatial incongruencies, which is in line with the current findings. However, I think the authors should interpret their negative result with more caution. The p-value was p=0.207 for the questionnaire data and p=0.244 for the proprioceptive drift data. And the N is small. Therefore, a false

negative result cannot be excluded. I assume that a Bayesian analysis would not provide strong evidence in favor of the null hypothesis.

Thank you for pointing out this useful comment. The paper you mentioned has been cited on page 17/18 of the manuscript as follows: "Altogether these findings suggest the DMI, like the somatic rubber hand illusion (Aimola Davies et al., 2013), is relatively robust to purely spatial incongruencies. Consequently, the mere synchronisation of tactile stimulation appears to be not only a necessary, but also a sufficient condition in order to produce changes in the spatial localisation and in the sense of ownership of teeth."

According to your suggestion, a Bayesian Analysis has been computed for the proprioceptive drift and the subjective rating in experiments 1-5 and the results of such analysis are reported in the supporting information section. Two sentences have been added to the Results section of the manuscript on pages 14 and 15 as follows:

p.14: "In addition to classical frequentist statistics, Bayesian repeated measures ANOVAs and Bayesian paired samples t-tests were also computed on the proprioceptive drift. Results of these analyses are reported as supporting information."

p.15: "In addition to classical frequentist statistics, Bayesian repeated measures ANOVAs and Bayesian paired samples t-tests were also on the rating for the DMI questionnaire's statements in experiments 1-5. Results of these analyses are reported as supporting information."

The reviewer is right in suggesting more caution when interpreting the results of experiment 3. Consequently the following sentence has been added on page 18 of the manuscript: "However, caution is required in interpreting these data as the Bayesian ANOVAs conducted in Experiment 3 provide only moderate evidence in support of the null hypothesis, namely that proprioceptive drift and subjective rating to statement 1 of the DMI questionnaire are independent of purely spatial modulation of the stroking stimulation.".

(ii) Regarding original point 4. I agree that the basic results that support the existence of the DMI are robust. The metanalyses presented in Figure 7 also nicely corroborates your main conclusion.

However, some of your secondary results could be false negatives; and here the small sample size in the individual experiments becomes a weakness. In particular, I am thinking about the negative findings in Exp 4 and 5 and the conclusion that you presented in the abstract: "DMI was not strongly affected by manipulating the macrogeometric or microgeometric tactile properties of the dental model, suggesting cognitive images of one's own oral morphology play a minimal role". However, the stroking x microgeometry interaction gave a p-value of 0.093, and in Figure 5D it looks like you have a positive result: the drift looks much greater in the congruent synchronous condition compared to the incongruent synchronous condition. Moreover, the questionnaire results for illusion statement 1 (Table 5) specifies a

main effect of macrogeometry at p=0.03, and a stroking x macrogeometry interaction of p=0.107. These are not the kinds of results that support a genuine negative finding in my opinion. If you want to present the negative macrogeometric result as a major finding of the study and highlight it in the abstract you should report a Bayes factor in favor of the null hypothesis using a Bayesian statistical analysis.

In addition to this concern, I think your macrogeometric manipulation is quite different from Tsakiris' block of wood condition. You have only removed one tooth, and that would be similar to removing one finger from the rubber hand I would imagine. So, the "top-down representation" effect in your paradigm is probably weaker compared to the classic block of wood condition. This would make the effect you are looking for small, which in turn implies that that experiment is probably underpowered. In sum, I think you should tone down your conclusions regarding a lack of an effect of macrogeometric incongruence and remove this result from the abstract.

I think the same goes for your strong conclusions regarding microgeometric incongruence in the discussion on page 61: "We conclude that surface texture is not a major modulator of tooth ownership". You should probably be more cautious here given the small N, the lack of Bayesian analysis favoring the null hypothesis, and the fact that not all results point in this direction. Look at the questionnaire result for example. In Table 2, the effect of microgeometry on illusion strength is p=0.080.

Thanks for encouraging additional caution in the interpretation of results observed in experiments 4 and 5. We have modified the text of the discussion section (pages 20-22) as follows:

"Bayesian analysis confirmed that the best model of the proprioceptive drift did not include a main effect of microgeometry. On the other hand, microgeometry is included in the most explanatory model of teeth ownership. However, this analysis provides moderate evidence against the exclusion of microgeometry in a model of proprioception and teeth ownership. We conclude that surface texture (Yoshioka & Zhou, 2009) is not a major modulator of tooth ownership. These findings are in line with the RHI literature, which shows that RHI does not depend on conceptual interpretation of tactile sensations (Schütz-Bosbach et al., 2009). People commonly sample the surface texture of the teeth with the tongue, particularly after eating, drinking or sleeping. However, our results indicate that these textural aspects exert only a minimal influence on the representation of the teeth themselves. We speculate that microgeometric textural modulations are attributed to external stimuli, such as food particles, that adhere to the tooth surface, rather than to one's own body.

Macrogeometric haptic perception of object shape requires extensive somatosensory processing, subsequent to simple somatosensory encoding (O'Sullivan et al.,1994). Therefore, experiment 5 sought to investigate the effects of macrogeometric similarity on the DMI. We replicated the trend from experiments 1, 2 and 4, with stronger proprioceptive drift in synchronous conditions. Interestingly, the changes due to synchronous stroking showed a tendency to depend on the structure of the dental model. When the dental model used in the paradigm had a missing incisor, proprioception (but not the explicit judgements of teeth ownership) showed a trend towards a reduced DMI. Bayesian analysis showed that macrogeometry played a role in explaining both

proprioceptive drift and explicit judgements of teeth ownership. This suggests some top-down influence of a cognitive dental model that includes macrogeometric features. The comparison of an internal model of our participants' complete dentition, and the finger's tactile experience of a tooth missing from the model would produce a conflict, so that what is felt on the teeth and with the finger no longer matches. The interaction between macrogeometry and synchrony achieved only trend-level significance, so caution is required in interpreting this result, particularly given the small number of participants. On the other hand, the direction of the interaction could be clearly predicted by previous studies of RHI. Lastly, no differences in rating emerged between stroking conditions for the average of the control statements for suggestibility. In conclusion, we find a modest evidence for a top - down, macrogeometric component of the DMI. We found no convincing evidence that a prior representation of tooth surface texture contributes to the sense of ownership with respect to one's own teeth. Conversely, we found moderate evidence that a macrogeometric model of dentition morphology could contribute to the sense of one's own teeth. We plan to replicate this latter result in further studies. Many people remember the strange and vivid experience of exploring with their tongue the gap left after a tooth falls out or is removed. Our results suggest interesting opportunities for longitudinal studies on updating internal models of one's own body before and after tooth loss."

Furthermore, the line of the abstract mentioning the results of experiments 4 and 5 has been modified as follows:

"DMI was moderately affected by manipulating the macrogeometric or microgeometric tactile properties of the dental model, suggesting cognitive images of one's own oral morphology play a modest role (Experiment 4 and 5)"

(iii) Regarding original point 11. Abdulkarim and Ehrsson (2016) observed a correlation between the sense of hand ownership and proprioceptive drift (shift) towards the rubber hand, but nevertheless, these authors urged caution when using the drift measure because they did not observe changes in hand ownership when they experimentally manipulated proprioception. Therefore, I suggest you reformulate your new sentence slightly:

"However, some studies have noted that this measure can dissociate from explicit reports, and have therefore recommend caution when using it (Abdulkarim & Ehrsson, 2016; Rohde et al., 2011)."

Thank you for your suggestion. The aforementioned sentence has been added to page 12 of the main text.

(iv) Regarding original point 21. Good that you have now included a few sentences about DMI's possible neural substrate in the general discussion. But I am only moderately impressed with your neuroanatomical speculations I have to admit. The posterior parietal cortex is certainly a good candidate, and you could add a couple of references at the end of the sentence where you mention this region (page 62): e.g., Ehrsson et al 2005a J Neurosci and Ehrsson et al. 2005b PLOS Biol. The intraparietal cortex of the posterior parietal cortex is active during the somatic rubber hand illusion (Ehrsson et al 2005a) and during a version of

Lackner's "Pinnochi illusion" induced by placing the vibration-stimulated hands against the waist eliciting "illusory waist shrinkage" (Ehrsson et al 2005b). Activity in the posterior parietal cortex also correlates with the proprioceptive drift towards the rubber hand (Brozzoli et al 2012 J Neurosci; a BOLD-adaption index of a shift in perihand space towards the rubber hand predicted the degree of proprioceptive shift towards the rubber hand). The somatic rubber hand illusion also activated the premotor cortex and cerebellum should I add. The premotor cortex could be relevant to mention because the activity in this region correlated with the strength of the self-touch illusion (Ehrsson 2005a). Moreover, in the brain of macaques, both the premotor cortex and the posterior parietal cortex contain extensive multisensory representations of the hand and mouth (Graziano and Cooke 2006 Neuropsychologia). The reasons for this is probably that hand and mouth actions are coordinated during eating behavioral, and that the hand is retracted towards the face during defensive action. Thus, in my opinion it is reasonable to speculate that these fronto-parietal regions could also be involved in the DMI. Regarding the Tsakiris et al (2007) Cerebral Cortex study, these authors reported a correlation between proprioceptive drift and rCBF in the posterior insula (in a fixed-effect analysis across four different conditions). This activation was not located in the secondary somatosensory cortex if I remember correctly (?); but PET has relatively low spatial resolution and posterior insula and secondary somatosensory cortex are adjacent regions that could be difficult to anatomically distinguish. More problematically, in the Tsakiris study it is not clear if the correlation is driven by the proprioceptive drift towards the rubber hand in the synchronous congruent (illusion) condition, as opposed to by differences in drift between the various control conditions. Buxbaum and Coslett (2001) analyzed structural descriptions of the body that involve semantic representation. This type of representation is probably different from the perceptual processes involved in the somato-proprioceptive illusion you are examining. Personally, I would not expect a common neural substrate between DMI and body structure description in the posterior parietal cortex.

Thank you for your comments. Some of the references you suggested have been added to page 23 of the manuscript as follows:

"**In fact, activity in these regions correlates with proprioceptive drift observed after RHI (Brozzoli, et al., 2012). This area also plays a critical role in representing spatial relations between body-parts (Buxbaum & Coslett, 2001). Lastly, premotor cortices could potentially be involved in DMI. Indeed, the strength of self-touch illusions has been shown to correlate with the activity in these regions (Ehrsson, 2005).**"

(v) New point 1. In Figure 3 what is plotted on the x-axis? I assume it is time but it would be nice to know the scale.

Figure 3 is not a graph. Rather, it is a scheme showing the different structure of experiments 1, 2 and 3. Consequently nothing has really been plotted on the "x-axis". The horizontal arrow in figure 3 represents the progression of the experiment over time (s).

(vi) New point 2. In Figure 3 it is difficult to read the individual statements. Please list the questionnaire statements in a supplementary table or make this figure panel bigger.

The reviewer is right in raising this point. A table listing all the individual statements of the Dental Model Illusion Questionnaire has been added to the supporting information section.
Moreover, a sentence has been added on page 8 of the manuscript as follows "<u>A list of all the statements of the DMI questionnaire can be found in the supporting information section.</u>"

(vii) New point. In Table 1 the title says "Experiments 1, 2, 4 and 5" but in the table Experiment 2 is not listed, but Experiment 3.

Thanks for pointing out this typing mistake. The title of table 1 has been updated.

(viii). Finally, in the previous review I forgot to ask you if you found a significant correlation between proprioceptive drift and questionnaire statement 1. You could compute the proprioceptive shift by subtracting the drift in the asynchronous condition from the drift in the synchronous condition, and then test if this shift correlates with the difference score in illusion strength (statement 1) when subtracting the asynchronous condition from the synchronous condition. You could run this analysis across all 32 subjects to enhance statistical power. In my opinion, observing a significant correlation between drift and subjective ratings would corroborate your main conclusion.

Thank you for raising this point. We computed the analysis you suggested, and the value of r is 0.002. The correlation between proprioceptive drift and subjective drift is therefore extremely weak and not statistically significant (p =.992).
We decided to not report this analysis in the main text because we did not predict any correlation between drift and subjective rating. Inserting that to the manuscript now would represent a mere post-hoc manipulation of the data collected.
